# Analysis of the SUMO2 Proteome during HSV-1 Infection

**DOI:** 10.1371/journal.ppat.1005059

**Published:** 2015-07-22

**Authors:** Elizabeth Sloan, Michael H. Tatham, Marine Groslambert, Mandy Glass, Anne Orr, Ronald T. Hay, Roger D. Everett

**Affiliations:** 1 MRC-University of Glasgow Centre for Virus Research, The Sir Michael Stoker Building, University of Glasgow Garscube Campus, Glasgow, Scotland, United Kingdom; 2 Centre for Gene Regulation and Expression, College of Life Sciences, University of Dundee, Sir James Black Centre, Dundee, United Kingdom; Baylor College of Medicine, UNITED STATES

## Abstract

Covalent linkage to members of the small ubiquitin-like (SUMO) family of proteins is an important mechanism by which the functions of many cellular proteins are regulated. Sumoylation has roles in the control of protein stability, activity and localization, and is involved in the regulation of transcription, gene expression, chromatin structure, nuclear transport and RNA metabolism. Sumoylation is also linked, both positively and negatively, with the replication of many different viruses both in terms of modification of viral proteins and modulation of sumoylated cellular proteins that influence the efficiency of infection. One prominent example of the latter is the widespread reduction in the levels of cellular sumoylated species induced by herpes simplex virus type 1 (HSV-1) ubiquitin ligase ICP0. This activity correlates with relief from intrinsic immunity antiviral defence mechanisms. Previous work has shown that ICP0 is selective in substrate choice, with some sumoylated proteins such the promyelocytic leukemia protein PML being extremely sensitive, while RanGAP is completely resistant. Here we present a comprehensive proteomic analysis of changes in the cellular SUMO2 proteome during HSV-1 infection. Amongst the 877 potentially sumoylated species detected, we identified 124 whose abundance was decreased by a factor of 3 or more by the virus, several of which were validated by western blot and expression analysis. We found many previously undescribed substrates of ICP0 whose degradation occurs by a range of mechanisms, influenced or not by sumoylation and/or the SUMO2 interaction motif within ICP0. Many of these proteins are known or are predicted to be involved in the regulation of transcription, chromatin assembly or modification. These results present novel insights into mechanisms and host cell proteins that might influence the efficiency of HSV-1 infection.

## Author Summary

Proteins are subject to many types of modification that regulate their functions and which are applied after their initial synthesis in the cell. One such modification is known as sumoylation, the covalent linkage of a small ubiquitin-like protein to a wide variety of substrate proteins. Sumoylation is involved in the regulation of many cellular pathways, including transcription, DNA repair, chromatin modification and defence to viral infections. Several viruses have connections with sumoylation, either through modification of their own proteins or in changing the sumoylation status of cellular proteins in ways that may be beneficial for infection. Herpes simplex virus type 1 (HSV-1) causes a widespread reduction in uncharacterized sumoylated cellular protein species, an effect that is caused by one of its key regulatory proteins (ICP0), which also induces the degradation of a number of repressive cellular proteins and thereby stimulates efficient infection. This study describes a comprehensive analysis of cellular proteins whose sumoylation status is altered by HSV-1 infection. Of 877 putative cellular sumoylation substrates, we found 124 whose sumoylation status reduces at least three-fold during infection. We validated the behavior of several such proteins and identified amongst them several novel targets of ICP0 activity with predicted repressive properties.

## Introduction

Herpes simplex virus type-1 (HSV-1) is an alphaherpesvirus which causes vesicular oral and genital lesions, and has the capacity to cause more severe diseases such as meningitis and encephalitis, particularly in immunocompromised individuals and neonates (see [1,2] for general reviews). Characteristic of an alphaherpesvirus, HSV-1 establishes latency within sensory neurons, from which reactivation occurs periodically. The lytic, latent and reactivation states are governed by the innate, intrinsic and adaptive immune responses and the mechanisms by which HSV-1 has evolved to counteract these immune responses.

The attachment and entry of HSV-1 into a cell causes the activation of the innate and intrinsic immune responses. The former involves production of interferons (IFNs) which activate signal transduction pathways, resulting in the expression of IFN stimulated genes (ISGs) (reviewed in [3]). Intrinsic antiviral resistance, on the other hand, is mediated by constitutively expressed proteins. Amongst the various factors that have been identified as contributing to intrinsic resistance are certain components of promyelocytic leukaemia (PML) nuclear bodies (PML NBs, also known as ND10), including the PML protein itself and other major components such as Sp100, hDaxx and ATRX [4]. Both PML and Sp100 are heavily modified by the SUMO family of ubiquitin-like proteins [5], and both sumoylation and interaction with sumoylated proteins are key factors in the assembly of PML NBs [6,7]. These proteins are recruited to sites of incoming HSV-1 genomes very early in infection [8] and they have the potential to restrict HSV-1 replication as soon as the cell becomes infected [9–12]. The mechanism of this recruitment is incompletely understood, but it is clear that both sumoylation and SUMO-mediated interactions play important roles [13].

The HSV-1 regulatory protein ICP0 reduces the sensitivity of HSV-1 to IFN [14–16] and also counteracts the restrictive effects of PML NBs through its ubiquitin E3 ligase activity [17]. ICP0 induces the degradation of the sumoylated forms of PML through an activity that has similarities to those of SUMO-targeted ubiquitin ligases (STUbLs) [18,19], and it also degrades the unmodified forms of the most abundant isoform of PML in a SUMO-independent manner [20]. In addition, at later times of HSV-1 infection a widespread loss of high molecular weight cellular SUMO conjugates occurs in an ICP0-dependent manner [18,19]. These activities combine to cause complete disruption of PML NBs, dispersal of those components such as hDaxx and ATRX that are not degraded, and inhibition of the recruitment of PML NB components and other as yet uncharacterized sumoylated proteins to the sites of HSV-1 genomes. Given that depletion of the only known SUMO E2 conjugating enzyme Ubc9 also diminishes intrinsic resistance to HSV-1 infection (and hence augments the replication of ICP0-null mutant HSV-1) [18], there is accumulating evidence that mechanisms that are regulated by sumoylation play an important part in intrinsic resistance to HSV-1 infection.

The SUMO family of proteins includes three members (for general reviews of the SUMO pathway, see [21,22]). By sequence, SUMO1 is 18% related to ubiquitin and is conjugated to specific lysine residues in substrate proteins through an isopeptide bond between its C-terminal glycine carboxyl group and the lysine side chain in the substrate. SUMO2 and SUMO3 are closely related to each other (and share about 50% identity with SUMO1) and they include an internal lysine residue to which other SUMO moieties can be conjugated, and hence they can form poly-SUMO chains. The SUMO conjugation pathway involves a sequence of events that are analogous to those of ubiquitin conjugation, with SUMO first forming a thioester bond with the SAE1/2 SUMO E1 activation enzyme, followed by the activities of Ubc9 and in some cases SUMO E3 ligases that catalyze conjugation to specific substrate proteins. Several previous studies have analyzed the diversity of cellular proteins that can be sumoylated under a variety of conditions [23–28].

In this study we set out to characterize changes to the cellular SUMO2 proteome in response to infection with wild type (wt) HSV-1 using Mass Spectrometry (MS)-based quantitative proteomics. Our experimental design allowed the identification of sumoylated proteins that are preferentially degraded in the presence of ICP0, and it also revealed a number of cellular proteins that were not candidate sumoylated species that were also degraded. We investigated a number of the identified proteins, including known controls such as PML and Sp100, to determine their sumoylation status and whether or not their degradation was ICP0 dependent. The data revealed widespread changes in the SUMO2 proteome during HSV-1 infection, revealing many proteins that are potentially involved in the regulation of gene expression that have hitherto not been previously identified or examined in the context of a viral infection. Of particular note, we identified several members of the ZBTB family of proteins, and other proteins with related BTB domains, that are susceptible to degradation by ICP0. We present examples of these proteins that are degraded by mechanisms that are influenced by modification by SUMO2/3, or on the presence of a SUMO2/3 interaction motif within ICP0. Our results provide detailed insight into the numerous and complex changes in protein stability that occur during HSV-1 infection.

## Results

### A system for SUMO proteome analysis of HSV-1 infected cells

**Fig 1:**
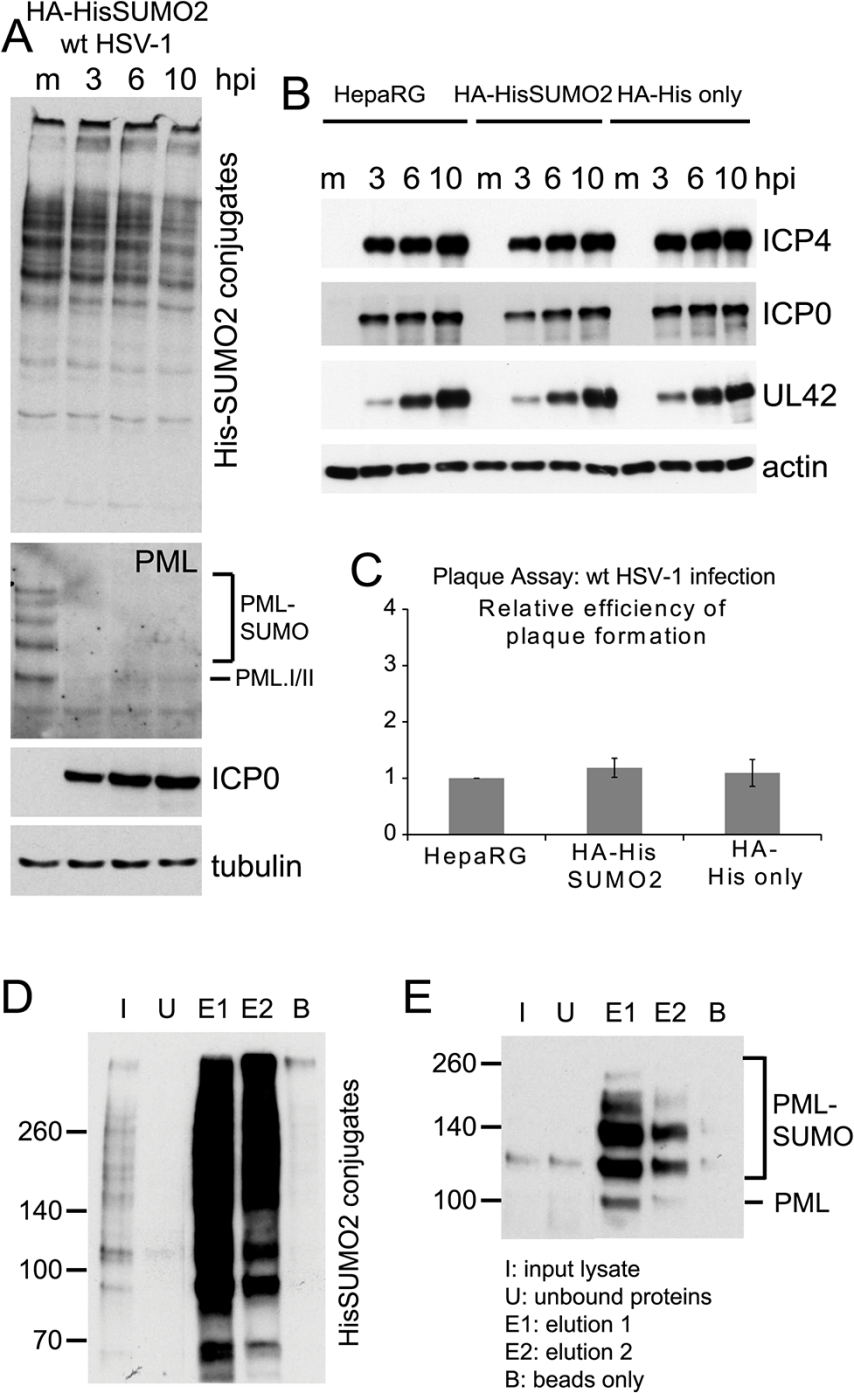
Characterization of HA-HisSUMO2 cells. (A) Cells expressing His-tagged SUMO2 were infected with wt HSV-1 at MOI 10 and samples were harvested at 3, 6, and 10 h p.i. Proteins were analyzed by western blotting using 6xHis, PML 5E10, ICP0 11060, and tubulin antibodies. Mock infected HA-HisSUMO2 cells were included as a control. (B) Kinetics of HSV-1 protein expression are equivalent in HA-HisSUMO2, parental HepaRG and HA-His only cell lines. Cell extracts were analyzed by western blotting for viral proteins ICP4, ICP0 and UL42, following wt HSV-1 infection for 3, 6, and 10 h (MOI 10). Mock infected controls (m) were included for each cell line and blots probed for actin as a loading control. (C) Plaque forming efficiency of wt HSV-1 in HA-HisSUMO2 cells relative to the parental HepaRG cell line. Data from replicate experiments was averaged, normalized to the HepaRG cell data and represented as mean +/- standard deviation. (D) His affinity purification of His-SUMO2 conjugated proteins. Samples collected throughout the affinity purification were analyzed by western blot for purification and elution of His-SUMO2 proteins. I–initial extract; U–unbound fraction; E1 and E2 –sequential elution samples; B–a sample of the remaining beads after elution. 1% of the initial lysate, 16% of total elution fractions and 4% of the bead fraction were loaded. (E) The same samples were analyzed for the presence of sumoylated PML.

Cells expressing poly-histidine tagged SUMO1, -2 or -3 were isolated after transduction with lentivirus vectors. HepaRG hepatocytes were used because they are readily infected by HSV-1 and, unlike human diploid fibroblasts (HFs), their growth was not compromised in isotopically labeled SILAC medium. We concentrated on poly-histidine tagged SUMO2 (His-SUMO2)-expressing cells (herein named HA-HisSUMO2) because of the potential for poly-SUMO2 chains and because of the existing depth of knowledge of the SUMO2 proteome [23–28]. Infection of HA-HisSUMO2 cells with wt HSV-1 caused a reduction in the overall abundance of His-SUMO2 conjugated proteins (Fig 1A), which was more marked at later times of infection (S1A Fig). This was not as pronounced as in previous studies using HFs [18], perhaps because of over-expression of His-SUMO2 (S1B Fig), or because reductions in overall sumoylation levels are less pronounced in HepaRG cells compared to HFs [18]. Despite the increased expression of SUMO2, the sumoylated forms of PML were not substantially more abundant in HA-HisSUMO2 cells (S1C Fig) and they were readily degraded by HSV-1 (Fig 1A). Wt HSV-1 gene expression (Fig 1B) and plaque formation efficiency (Fig 1C) were as efficient in HA-HisSUMO2 cells as in parental HepaRG cells and control transduced cells expressing the His-tag only (HA-His only cells). Surprisingly, the plaque formation efficiency of ICP0-null mutant HSV-1 was increased in HA-HisSUMO2 cells (S1D Fig). Given previous results on the role of Ubc9 in restricting this virus [18], it might be expected that over-expression of SUMO2 could be inhibitory. On reflection however, this result does not necessarily challenge the hypothesis that sumoylation of repressive proteins, and/or their interactions with sumoylated proteins, contributes to the regulation of HSV-1 infection. Over-expression of a protein can affect the proper functioning of the pathway in which it is involved, and in this case over-expression of SUMO2 might affect the balance of interactions between sumoylated proteins and those containing SUMO interaction motifs (SIMs). Support for the role of sumoylation in intrinsic resistance to HSV-1 infection comes from a study of HepaRG cells highly depleted of SUMO2/3, in which PML NBs are disrupted and ICP0 null mutant HSV-1 replicates with increased efficiency (M. Glass, manuscript submitted for publication). These issues are clearly open for further experimentation, but they are beyond the scope of this particular paper.

### Experimental design of SUMO2 proteome analysis

**Fig 2:**
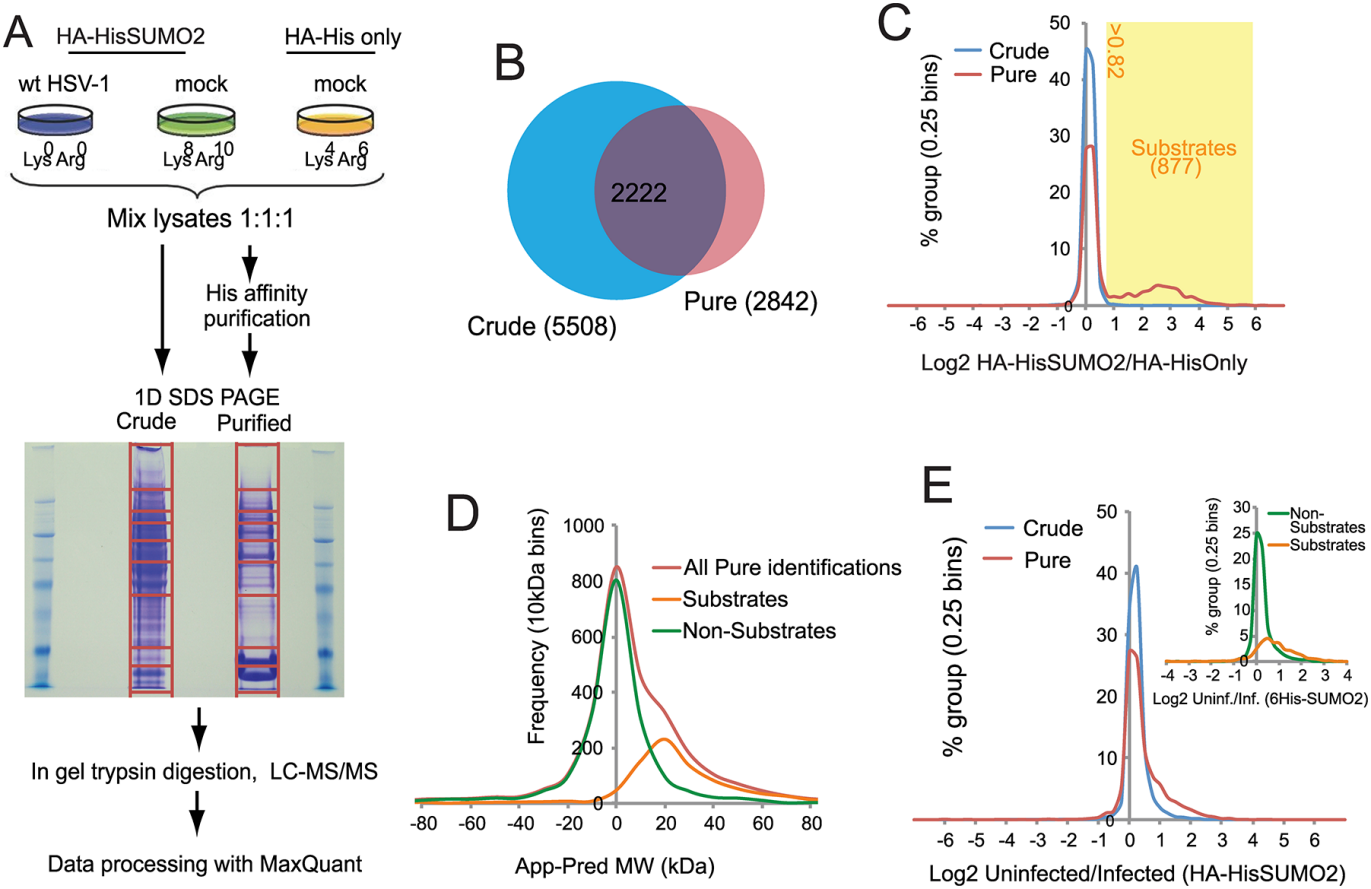
Overview of SILAC/Mass Spectrometry proteomics analysis comparing mock and wt HSV-1 infected HA-HisSUMO2 cells. (A) HA-HisSUMO2 cells were maintained in either SILAC L or H media (see Materials and Methods) and HA-His only cells were maintained in SILAC M medium. Cells maintained in L medium were infected with wt HSV-1 at MOI 10 for 12 h prior to lysing cells from all 3 treatments under denaturing conditions. Equal amounts of each lysate were mixed and subjected to nickel affinity purification. Proteins were then separated by SDS-PAGE, with the gel sliced into the sections for in gel tryptic digestion, as indicated. Samples were then analyzed by high resolution LC-MS/MS with data processed using MaxQuant software. (B) Comparison of the number of protein IDs in the crude and purified fractions, and the degree of overlap. (C) A frequency chart of protein IDs in the crude and purified fractions was plotted against log_2_ of their H/M ratios, revealing a shoulder of 877 likely sumoylated species with a log_2_ ratio value >0.82. (D) Gel slice analysis of putative sumoylated and non-sumoylated substrates. Average apparent (App) molecular weights of proteins IDs were deduced from the gel slice of isolation and compared to their predicted (Pred) molecular weights based on sequence. This analysis distinguishes likely non-substrates (with a normal distribution around a App-Pred value of zero) from likely substrates distributed around a peak App-Pred value of about 20. (E) Changes in H (uninfected)/L (infected) ratios of proteins IDs in the crude and purified fractions, revealing a shoulder of proteins with increased H/L ratios. The inset shows a similar analysis of putative sumoylation substrates compared to non-substrates in the purified fraction.

A method involving nickel affinity purification of His-tagged sumoylated proteins under denaturing conditions [27] gave efficient recovery of both overall sumoylated proteins (Fig 1D) and the specific example of sumoylated PML (Fig 1E). HA-HisSUMO2 cells were grown in isotopically normal (light; L) or heavy (H) SILAC media (in which lysine and arginine have the heavy isotopes of nitrogen (^15^N) and carbon (^13^C)). Uninfected HA-His only control cells were grown in isotopically intermediate (M) medium, as defined in the methods section. The L cells were infected with wt HSV-1 at MOI 10, then all but one plate of cells from each condition were harvested directly into guanidinium denaturing buffer 12 h later. The H, L and M lysates were mixed in equal protein amounts then used for nickel affinity purification of SUMO2-modified proteins. To allow analysis of total protein abundance changes and confirm efficient virus infection and degradation of previously characterized cellular proteins, cells from one plate of each set were harvested directly into SDS-PAGE loading buffer. Samples of the crude and affinity purified mixtures were subjected to SDS-PAGE, then the gels were stained and cut into slices for in gel tryptic peptide production. The peptides were analyzed by LC-MS/MS and the data analyzed using MaxQuant software (See Materials and Methods for details). Fig 2A shows a flow diagram of the experimentation and images of the resulting SDS-PAGE stained gels. Data derived from the crude and purified samples gave information on the relative levels of total protein and putative sumoylated protein species respectively. Analysis of the H/M ratios in the purified sample allowed the separation of likely sumoylated species from non-specifically purified proteins, while the H/L ratios of these protein IDs in the same sample can be used to assess changes in relative abundance during HSV-1 infection.

### Designation of putative SUMO2 substrates

A total of 6128 cellular proteins were identified, with 5508 and 2842 from the crude and purified fractions respectively, of which 2222 were in common (S1 Table, sheet 1 and Fig 2B). A number of proteins were detected only in the affinity purified sumoylated fraction, probably because their intrinsic abundance is too low for detection in the crude (Fig 2B). Because the majority of proteins would not be expected to change in abundance during infection, and also the presence of non-sumoylated proteins in the purified fraction, MaxQuant internally calculated normalised ratios could be used to correct for any errors in the mixing of the various samples prior to gel electrophoresis. Frequency plots for log_2_ HA-HisSUMO2 to HA-His only ratios (log_2_ H/M) showed little variation in abundance of proteins in crude extracts (Fig 2C, blue line), with the frequency plot forming a tight normal distribution around the 1:1 ratio region. Two distinct sub-populations can be seen by the same analysis of data derived from purified samples (Fig 2C, red line). While there is a large peak of proteins with ratio 1:1 (log_2_ = 0), consistent with these being non-specific purification contaminants, there is also a smaller, broader peak in the region of log_2_ ratio of 1 to 6. These proteins are much more abundant in nickel purifications from HA-HisSUMO2 expressing cells compared to those from HA-His only cells, and so are likely to be SUMO2 conjugates. Ratio cut-offs for H/M and M/L were defined such that an estimated false discovery rate of less than 1% for SUMO2 substrates was applied (see Methods for further details), giving 877 putative SUMO2 conjugates (including multiple isoforms of some proteins such as PML), as listed (S1 Table, sheet 2). This method of SUMO2 substrate identification was validated by assessing the difference between the apparent MW of proteins based upon gel retention, and their predicted MW by sequence alone (Fig 2D) (see [29] for details of the method). The substrates and non-substrates clearly form two independent distributions in frequency plots, with substrates running in gels on average 20 kDa heavier than expected. Furthermore, comparison with a previous SUMO2 proteome analysis at the level of identified modification site [26] indicated 324 proteins in common with this set, while comparison with several major SUMO2 proteome studies revealed 521 proteins in common [23–28] (S1 Table, sheet 2, column P). In summary we can be confident that this list of 877 proteins represents true cellular SUMO2 substrates under these experimental conditions. A complete listing of all the data on the cellular proteins identified is presented in S1 Table. In addition to these cellular proteins, 71 viral proteins (i.e. all but one of the major viral polypeptides, the exception being US5) were identified in the crude sample (S2 Table).

### The effect of HSV-1 infection upon the SUMO proteome

H/L ratios can be used to study changes to either the total proteome (via ‘crude’ data), or the SUMO proteome (via ‘pure’ data) upon HSV-1 infection. Frequency distribution charts comparing ‘crude’ ratios from infected cells and uninfected cells showed few changes in total protein levels (Fig 2E, blue line). However, while most proteins in purified preparations also showed no change in abundance, the distribution is skewed towards larger ratios (Fig 2E, red line), with putative SUMO2 substrates being mostly responsible for this high ratio tail (Fig 2E, insert). This shows that the non-substrates are largely unchanged during HSV-1 infection, while SUMO2 conjugates have a tendency toward high ratios, indicative of a widespread loss of SUMO2 conjugation during infection. This is consistent with western blot data (Fig 1A). To test the reproducibility of these data a similar quantitative proteomic experiment was undertaken, this time only including Light infected and Heavy uninfected samples (S2A Fig). Although the total number of proteins was lower in this compared to the triple labeled experiment (S2B Fig), there was considerable overlap between the proteins in the purified fractions (S2C Fig), and the details of their ratio changes correlated substantially (S2D Fig; S3 Table). Because the triple labeled experiment was both larger and included the His-only control, subsequent sections mostly refer in detail only to this dataset.

**Fig 3:**
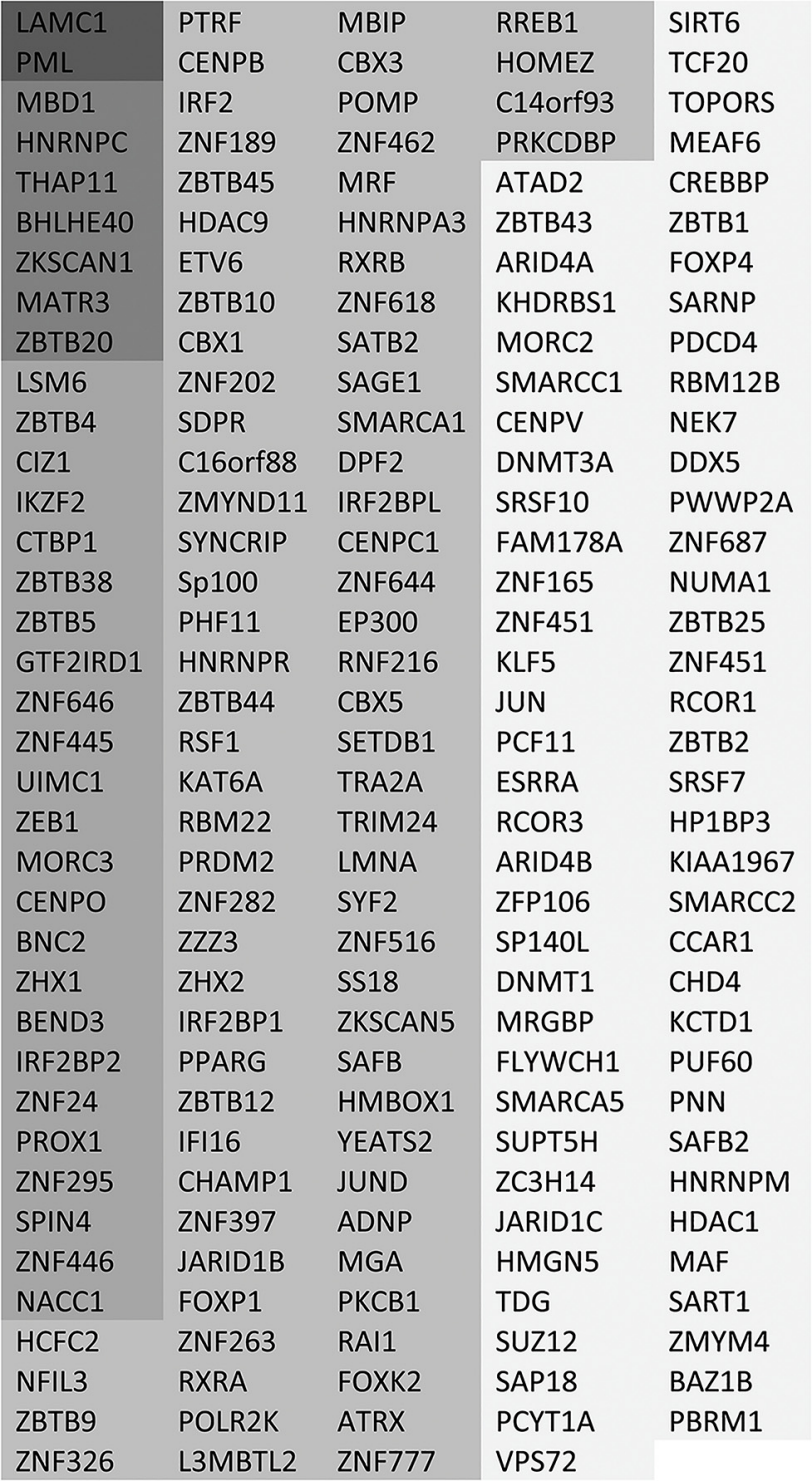
List of putative SUMO2 substrate proteins that are reduced in abundance in the purified infected compared to uninfected samples. The entries are shaded by degree of change, and all gave H/L ratios of 2 or greater with SigB values <0.1. The darkest shading indicates greater than 10-fold decrease, then in order of shading 7–10 fold, 5–7 fold, 3–5 fold and 2–3 fold.

To determine which SUMO2 substrates changed significantly during HSV-1 infection, Significance B (SigB) values were calculated using Perseus from the MaxQuant suite of software [30]. SigB is calculated using both signal intensity and SILAC ratio, and is indicative that a protein abundance change significantly deviates from the bulk of the quantified proteins. Of the 877 putative sumoylated proteins, 260 changed in abundance (either up or down) in the purified fraction of the triple SILAC experiment with SigB values of less than 0.1 (S1 Table, sheet 3). This number includes duplicate entries for proteins, such as PML, for which more than one isoform was detected. Removal of these duplicates and restricting the list to entries with H/L ratio increases of 2-fold or more results in a list of 185 proteins, shaded according to degree of change, and listed in order of decreased abundance (Fig 3). An additional 18 proteins on the putative sumoylated substrates list were detected with H/L ratios of 2 or greater and SigB values of less than 0.1 in the purified fraction of the replicate experiment, but which were not recorded as significantly regulated substrates in the primary experiment (S4 Table). As shown below, at least one of these is an authentic sumoylated substrate whose abundance decreases during HSV-1 infection. Overall therefore, up to around 200 cellular proteins identified in the purified fractions of the experiments decreased significantly in abundance during infection.

The degree of change in abundance of the putative sumoylated forms of these proteins varies considerably, up to a maximum of greater than 20-fold. In broad view, these data are consistent with previous observations on the decreased stability of sumoylated cellular proteins during HSV-1 infection. They also support the idea that there is considerable specificity or selectivity to the extent of sensitivity to desumoylation, as two-thirds of sumoylated proteins remain largely unaltered during infection while only 14% and 1% decrease in abundance by over 3-fold and 7-fold respectively.

We also identified a further 72 proteins that were not defined as potentially sumoylated on the basis of H/M ratios, which nonetheless had H/L ratios of greater than 2 and SigB of less than 0.1 in the purified fraction (S5 Table), although only 32 of these also complied with these criteria in the double labeled experiment. These may represent proteins that are non sumoylated, but which have an affinity for the Ni-agarose beads and whose abundance decreases during HSV-1 infection.

A small number of putative sumoylated proteins listed in S1 Table sheet 3 also exhibited H/L ratios of greater than 2 in the crude fraction (S6 Table), indicating candidate SUMO2 substrates whose total protein levels also reduced in infected cells. In the cases of IFI16, CENPB and PML, this has been reported previously [19,31–33]. Another protein on this list (NACC1) will be considered below, and further data presented below suggests that this list does not include all proteins that behave in this manner.

The levels of a number of proteins were changed significantly in the crude samples (S1 Table, sheet 4). Of these, and excluding those already noted above as regulated SUMO2 substrates, 128 proteins gave H/L ratios of greater than 2 in the crude samples (S7 Table). Given that HSV-1 infection causes the shut-off of host protein synthesis through induction of mRNA instability [34], this list is perhaps shorter than might be expected. It is possible that low abundance proteins with short half-lives (and thus those most susceptible to decreased host transcription) have not been detected efficiently in the ‘crude’ preparations by this approach, which will naturally favor the most abundant cellular proteins.

### Cellular proteins which exhibit apparently increased sumoylation or increased abundance during HSV-1 infection

An unexpected finding concerns a small group of cellular proteins whose degree of sumoylation appears to contradict the general trend of deconjugation, and actually increases during infection. S8 Table shows proteins with H/L ratios in the purified fraction of less than 0.5 and SigB values of less than 0.1. Several of these proteins are components of the basic transcriptional apparatus (MED9, TAFs 1, 9 and 12) or transcription factors (MAFA, MAFB). This may indicate overall changes in transcription complexes as infection progresses. Validation of an example of a protein in this category (ZBTB7A) will be presented below. Similarly, in the crude fraction a small number of cellular proteins increased in overall abundance by a factor of 2-fold or more and with SigB values of less than 0.1 (S9 Table). Perhaps surprisingly, no induction of interferon-stimulated genes was detected under these infection conditions.

### Analysis of viral proteins

**Fig 4:**
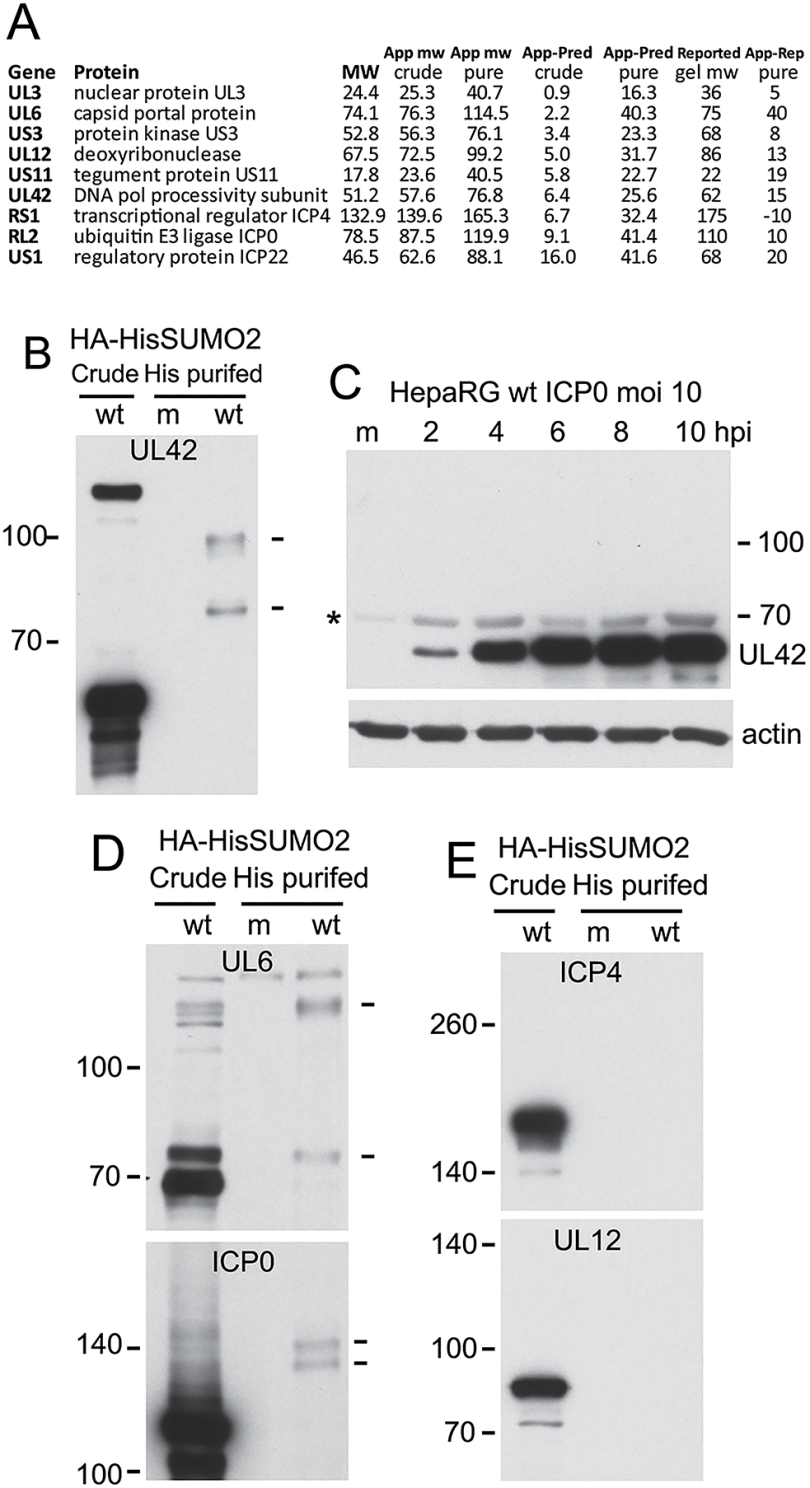
Analysis of potential sumoylation of HSV-1 proteins. (A) Tabulation of the nine HSV-1 proteins (excluding glycoproteins) whose apparent molecular weights in the purified fraction exceed those in the crude. App and Pred MW indicate apparent (based on gel slice data) and predicted (based on primary sequence) molecular weights, while reported gel MW indicates the established apparent MW based on gel mobility as extensively reported in the literature. (B) Western blot analysis of UL42 in crude and purified HA-HisSUMO2 fractions of cells infected with wt HSV-1 (MOI 10 at 12 h after infection; m indicates a purified mock infected sample prepared in parallel), showing probable sumoylated species in the purified infected sample (indicated by dashes on right). (C) Sumoylated forms of UL42 are below the level of detection in total protein extracts of wt HSV-1 infected HepaRG cells. The asterisk on the left indicates a non-specific background band. (D) The samples used in B were analyzed for UL6 and ICP0. Trace amounts of potential sumoylated forms of UL6 and ICP0 are indicted by dashes on the right. (E). The same samples were analyzed for ICP4 and UL12.

By applying the same method for monitoring apparent in gel molecular weights of cellular proteins (see Fig 2D) we were able to investigate the difference between apparent and predicted molecular weights of viral proteins. Most viral proteins were found in gel slices that were consistent with their predicted molecular weights, although the UL26 capsid maturation protease exhibited higher gel mobility than expected by sequence alone, consistent with its known cleavage during capsid assembly and also the production of its C-terminal half (protein VP22a) as a separate protein from an independent transcription unit [35,36]. Several proteins, however, had lower gel mobilities than predicted. In the case of glycoproteins gC, gL, gK and gM this is likely due to glycosylation (S2 Table). A small group of viral proteins exhibited decreased gel mobility over that predicted in the purified but not the crude sample, and in most of these cases the size difference could be consistent with sumoylation (Fig 4A). Note that the predicted molecular weight of some of these proteins differs considerably from their established gel mobilities, and in the case of ICP0, for example, this has been attributed to the nature of the primary sequence rather than post-translational modification. Therefore we investigated whether putative sumoylated species could be detected by western blotting of purified extracts from infected HA-HisSUMO2 cells.

Analysis of UL42, the processivity factor for the viral DNA polymerase, in the crude and purified samples of infected HA-HisSUMO2 cells revealed clear evidence of slower migrating species whose mobility is consistent with sumoylation (Fig 4B). This is of interest because the analogous protein (UL44) of HCMV is sumoylated [37]. However, these putative sumoylated UL42 bands were not clearly detectable in the crude fraction of HA-HisSUMO2 cells, nor were they evident during a normal wt HSV-1 infection of control HepaRG cells (Fig 4C). It is possible that over-expression of SUMO2 in the HA-HisSUMO2 cells forces a sumoylation event, or shifts the sumoylation equilibrium so that such species become more detectable. Therefore, while the evidence indicates that sumoylation of UL42 can occur, the likely sumoylated species seem to be in very low abundance during the course of a normal infection. We also detected likely sumoylated forms of UL6 and ICP0 in the purified fraction (albeit for the latter only on very long exposures of the blot) (Fig 4D), and extended exposure of the UL12 samples also revealed a possible sumoylated form. For the other proteins on the list of Fig 4A, we were either unable to detect sumoylated species by western blot of purified fractions (Fig 4D), or we lacked the reagents required to perform the analysis. Considering the scale of the proteomic method, it is conceivable that the sensitivity of the mass spectrometric approach is higher than the western blots shown here, and the possibility that all these proteins have a sumoylated component cannot be excluded. Analysis of the sequences of these proteins for potential sumoylation sites revealed consensus modification sites in US3, UL12 and UL42, but not the others.

### Pathway and protein family analysis of proteins that significantly alter in abundance during HSV-1 infection

**Fig 5:**
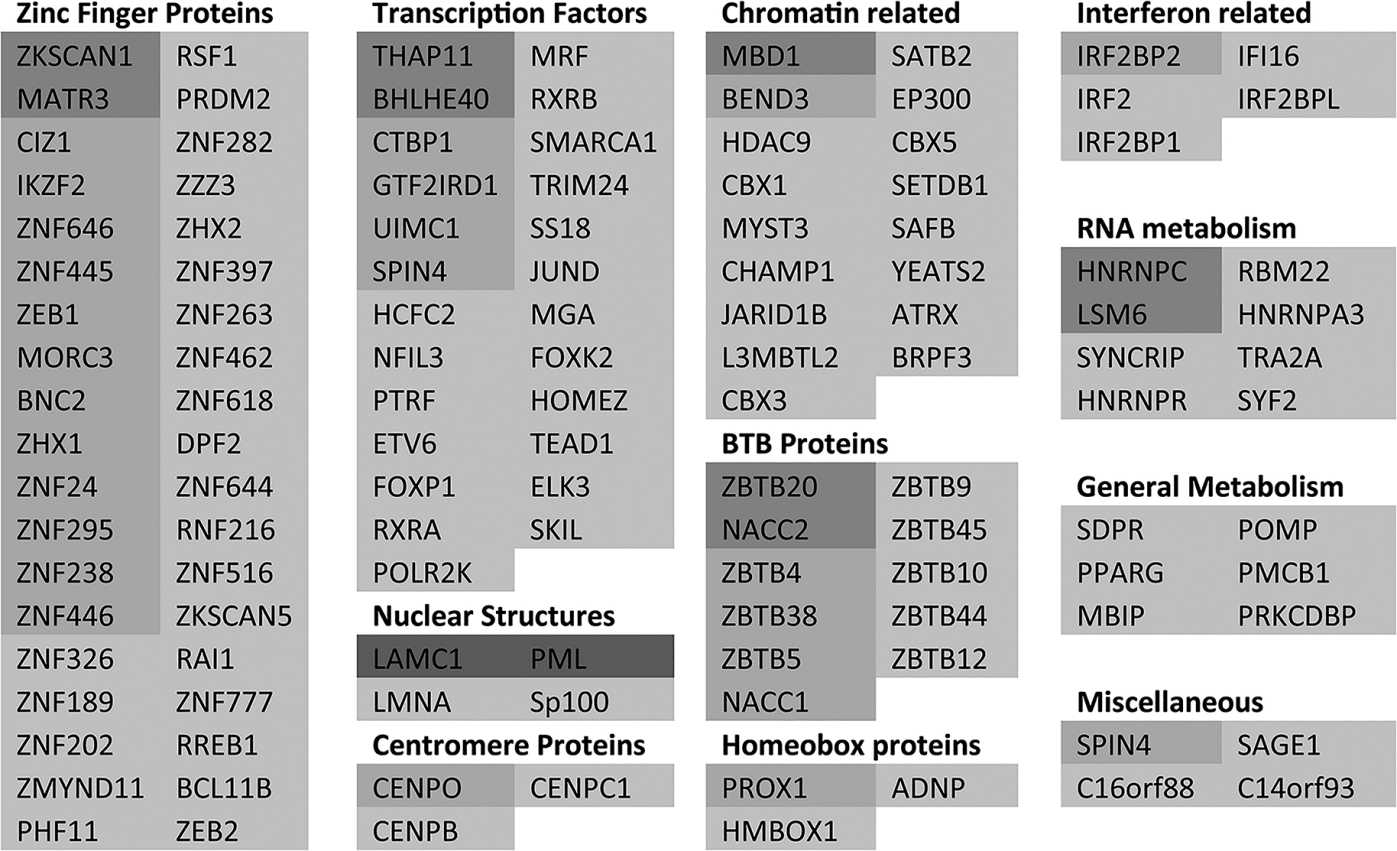
A listing of the proteins showing greater than 3-fold increases in H/L ratios. The proteins are taken from entries in Fig 3 and S4 Table, grouped according to general function or characteristics and shaded according to degree of ratio change (as used in Fig 3). Asterisks indicate proteins from S4 Table. There is some overlap between some of the categories, for example many BTB proteins also have a zinc finger, while many zinc finger proteins could equally be described as transcription factors or chromatin related.

As an initial step towards functional analyses of the proteins showing the greatest degree of change in sumoylation during HSV-1 infection, we grouped the 124 proteins of Fig 3 and S3 and S4 Tables with greater than 3-fold increases in H/L ratios in the purified fractions (pooling the data from experiments 1 and 2), then grouped them into broad, sometimes overlapping, categories (Fig 5). The proteins in each category are listed in order of degree of H/L ratio change and shaded as in Fig 3. The largest group of proteins include those with zinc finger domains, followed by transcription factors and chromatin-related proteins. BTB proteins, many of which have an additional zinc finger domain (the ZBTB proteins), form another marked group. There is a group of nuclear structure components such as lamins, and the PML NB components PML, Sp100 and MORC3, and also three centromere proteins. Several centromere proteins are already known to be degraded during HSV-1 infection in an ICP0-dependent manner [33,38,39]. Other groups of proteins have functions in RNA metabolism, interferon related pathways, and general metabolism (such as kinases).

It is striking that so many ZNF and ZBTB proteins were identified as regulated substrates, opening the question whether the zinc finger or the BTB domain of itself is contributing to the sensitivity of the sumoylated forms of these proteins to HSV-1 mediated degradation. We identified approximately 200 proteins with ZNF in the gene name (this will not include all proteins that include a zinc finger) of which 59 were sumoylated candidates and 24 of these were reduced in abundance by 3-fold or more. For ZBTB proteins, 19 in total were detected, 18 of which were sumoylation candidates and 11 were reduced in abundance by 3-fold or more. Thus the presence of a zinc finger or ZBTB domain itself does not generally confer sensitivity to HSV-1, but rather it seems that the proportion of these classes of proteins that are subject to sumoylation is increased compared to the bulk of cellular proteins.

Analysis of the functional consequences of these changes in abundance of these proteins during HSV-1 infection is obviously beyond the scope of this study, but the results certainly provide many novel avenues to pursue. Especially for those proteins undergoing the most dramatic changes in sumoylation, and in some cases overall abundance, there will inevitably be substantial disruption of the pathways in which they are involved during HSV-1 infection. Known examples of this include disruption of PML NBs and centromeres. But it is also reasonable to expect that the effects on chromatin related proteins and transcription factors will have consequences to chromatin structure or modification and transcriptional activity. Given that previous SUMO proteomic studies have highlighted that sumoylation of the proteins that are involved in these pathways is common [23–28], it is not surprising that these pathways feature prominently amongst those that are potentially disrupted by HSV-1 infection. While many such proteins may be innocent victims that are affected simply because of their sumoylation status, it is likely that this analysis includes previously unrecognized proteins which impact on the efficiency of HSV-1 infection.

### Validation of changes in selected cellular proteins during HSV-1 infection

**Fig 6:**
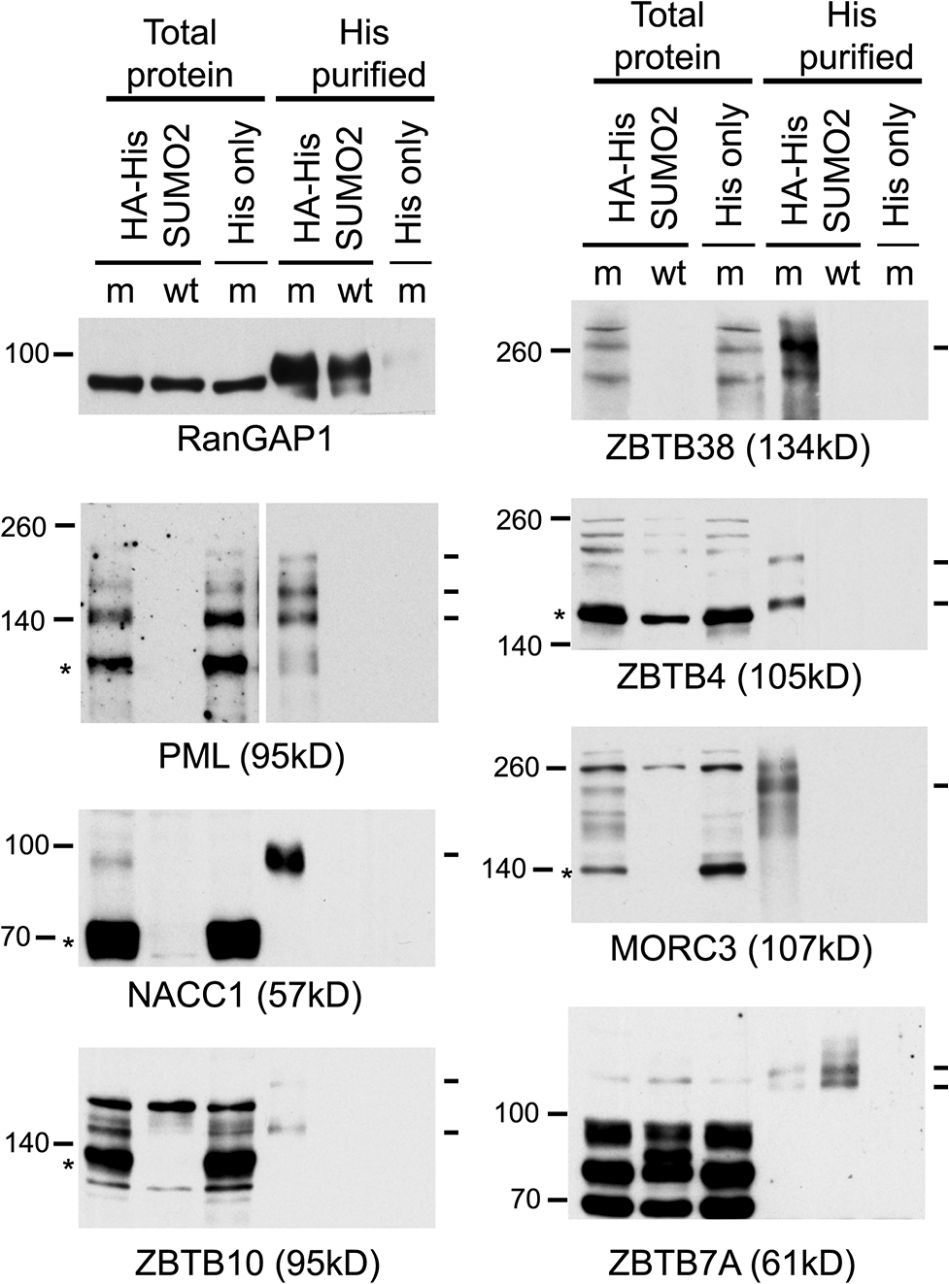
Confirmation that selected proteins of interest are present in His-SUMO2 purified samples and change in abundance during HSV-1 infection. Total and purified protein fractions from mock (m) and wt HSV-1 (wt; MOI 10 12 h p.i.) infected HA-HisSUMO2 cells were analyzed by western blot, with uninfected HA-His only total protein and purified fractions included as controls. Mock infected and infected samples are indicated by m and wt respectively. Probable sumoylated species of PML, NACC1, ZBTB10, ZBTB38, ZBTB4 and MORC3 were detected in the uninfected purified samples (indicated by dashes on the right of each panel), and were reduced in abundance during HSV-1 infection. ZBTB7A exhibited apparently increased SUMO2-modification in the infected purified fraction. RanGAP1 was used as an unchanged control for SUMO2-modification following HSV-1 infection. The predicted unmodified molecular weight of each protein is indicated in each panel, and where clear the likely major unmodified form is marked by an asterisk. For PML, this refers to the major isoforms PML.I and PML.II.

The proteomic data for several previously studied proteins were consistent with their established behaviour during HSV-1 infection. For example, the H/L ratios in both crude and purified fractions were increased for PML (for which both sumoylated and unmodified forms are known to be degraded [18,19]), while the Sp100 H/L ratio increased only in the purified fraction (consistent with the loss of only the sumoylated forms [40,41]), and there was no change in H/L ratio for RanGAP1 (which is neither degraded nor regulated at the level of sumoylation during HSV-1 infection [19]) (S1 Table, sheet 2; and S3 Fig). We analyzed a number of proteins listed in Fig 3 that had not been previously investigated, selected on the basis of being amongst those with the greatest changes in H/L ratios, or being representatives of groups of related proteins, and on antibody availability. ZBTB4, ZBTB10, ZBTB38, NACC1 and MORC3 all exhibited high H/L ratios in the purified sample, while NACC1 and to a lesser extent MORC3 and ZBTB4 also showed high H/L ratios in the crude samples, indicative of reduced total protein amounts (peptides for ZBTB10 and ZBTB38 were not detected in the crude sample). Total protein and His-purified extracts of uninfected and infected HA-HisSUMO2 cells and uninfected HA-His only cells were blotted for the above proteins, using PML and RanGAP1 as controls (Fig 6). The sumoylated forms of PML were readily identified in the purified sample of uninfected HA-HisSUMO2 cells, and both these and the major unmodified form were degraded during infection. In contrast, sumoylated RanGAP1 was stable (Fig 6, upper left panels). Similarly, a sumoylated form of NACC1 was detected, and both this and the non-sumoylated form were degraded. Analysis of the other proteins was complicated by likely non-specific bands, but in all cases bands consistent with sumoylated forms were detected in the purified fraction, and in all cases except ZBTB7A (see below) these were lost during infection. For ZBTB10 and MORC3 (and ZBTB4 to a lesser extent), likely unmodified forms (marked by asterisks) were also diminished during infection. The anti-ZBTB38 antibody was particularly prone to detection of potentially spurious bands, but likely sumoylated forms were clearly detected in the uninfected sample and lost during HSV-1 infection (Fig 6, upper right panel, see also below). Therefore, where reagents of sufficient quality are available, these results validate the SILAC data with a high degree of success. They also reveal a number of proteins whose apparently unsumoylated forms are also degraded during HSV-1 infection, and may therefore constitute previously unrecognised substrates of ICP0.

ZBTB7A was selected as a representative protein with a low H/L ratio in the purified fraction, potentially indicating an increase in abundance of sumoylated forms following infection. Potential sumoylated species of ZBTB7A were detected, albeit weakly, in the purified sample, and these were of increased abundance in the infected sample (Fig 6), again consistent with the proteomic data.

### Time course of degradation of selected cellular proteins during HSV-1 infection

**Fig 7:**
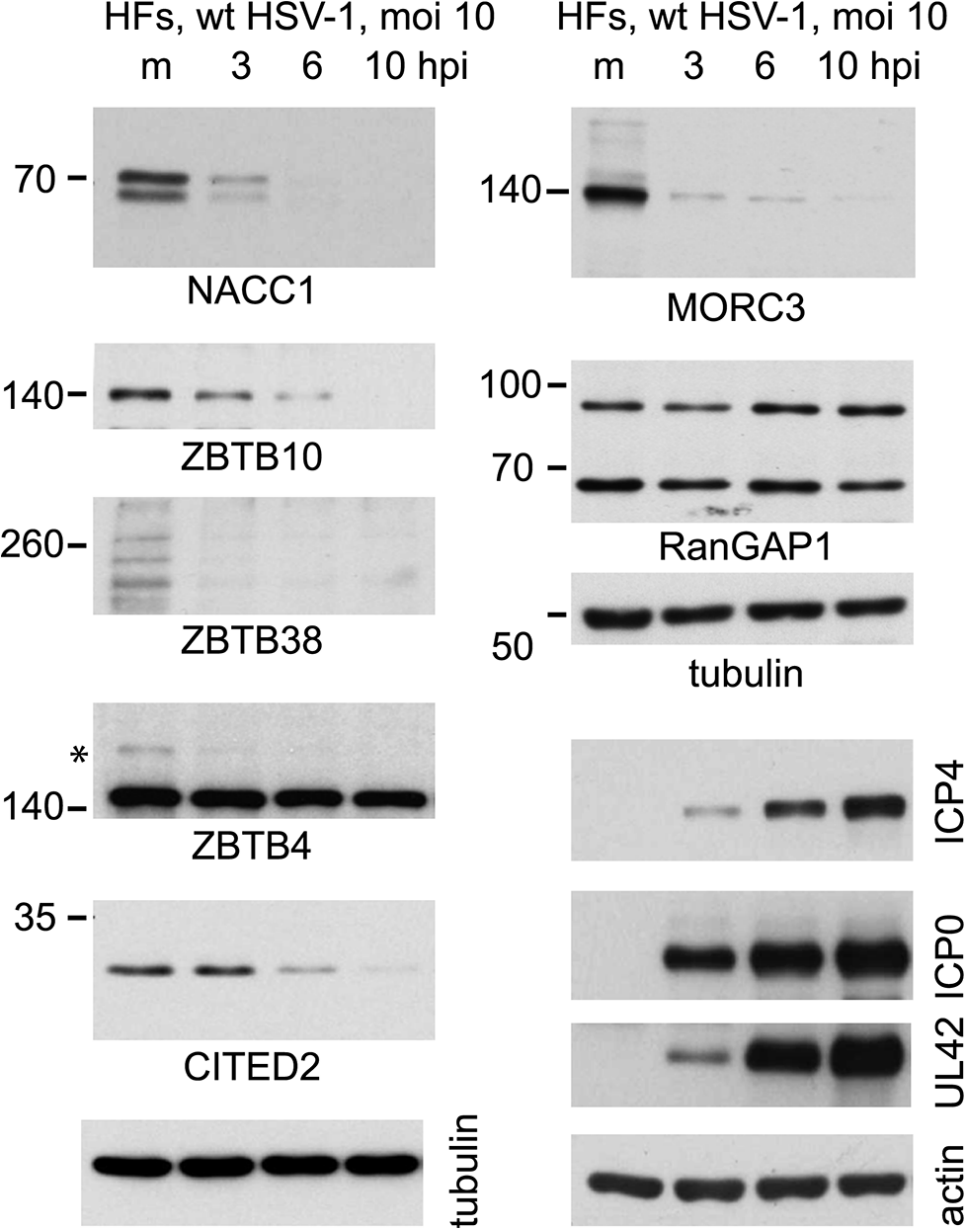
Time course of degradation of selected proteins of interest during wt HSV-1 infection. HFs were infected with wt HSV-1 (MOI 10) then samples harvested at 3, 6 and 10 h after infection were analyzed by western blot for the indicated proteins. For ZBTB4, the asterisk marks a potentially sumoylated species that decreases in abundance during infection. The panels on the lower right of the figure show the analysis of selected viral proteins in these samples.

By analyzing total protein extracts over time following HSV-1 infection of normal human fibroblasts, we found that the major forms of NACC1, ZBTB10, ZBTB38 and MORC3 were all degraded within 3 or 6 h (Fig 7). In these cells, the slower migrating sumoylated forms were not generally detected in the total protein extracts, although in the case of ZBTB4 the major form of the protein appeared stable while a potential sumoylated species was rapidly lost. The difference between the fate of the major form of ZBTB4 in this Fig compared to that in Fig 6 may be due to cell type, as the latter was performed in HA-HisSUMO2 cells. CITED2 was included in this analysis as an example of a protein with a high H/L ratio in the purified fraction, yet for which there was no evidence of authentic sumoylation (S1 and S3 Tables). This protein was also rapidly degraded during HSV-1 infection (Fig 7).

### Expression and characterization of further examples of potential sumoylated proteins

**Fig 8:**
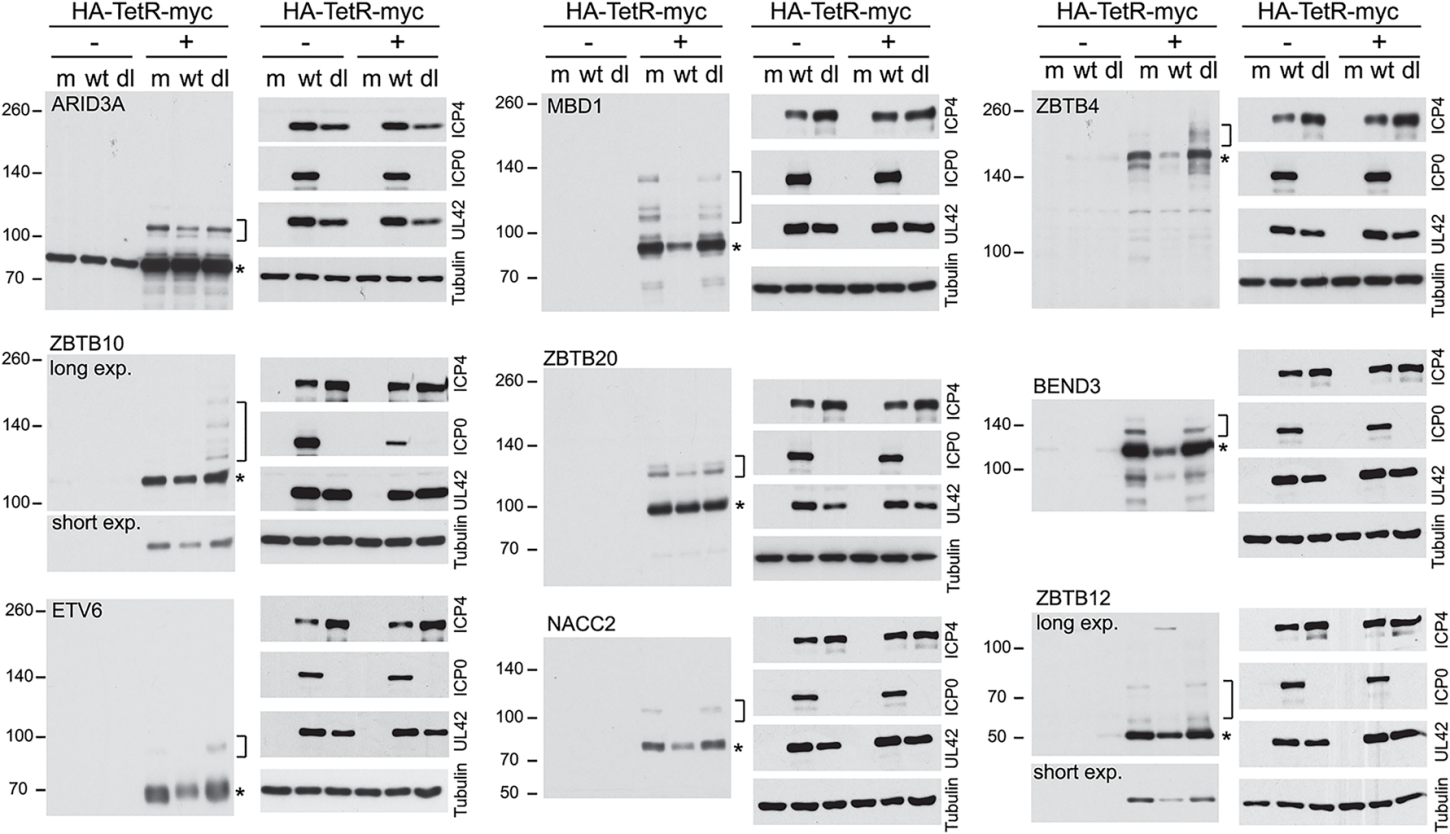
Degradation of selected sumoylated proteins in an ICP0-dependent manner during HSV-1 infection. HepaRG based cells were established that express the indicted myc-tagged proteins in an inducible manner. The cells were untreated (-) treated with doxycycline (+) for 2 h (1 h for ETV6, 24 h for ZBTB4), then the doxycycline was washed out and the cells infected with either wt or ICP0 null mutant (dl) HSV-1 (MOI 10 for 8 h) or mock infected (m). Whole cell lysates were analyzed using antibodies to detect the myc tag and the indicated viral proteins, with tubulin as the loading control. The asterisks and brackets indicate major likely unmodified and sumoylated forms of the proteins respectively.

While confidence in the reliability of the proteomic data is strengthened by the above results, the analysis is limited by the quality of available antibodies. Therefore we selected further candidate proteins for study using an inducible expression system [42]. This allows addition of an epitope tag and expression in a high proportion of transduced cells at levels that could be controlled by the length of time of induction. The proteins selected included some analyzed in Figs 5 and 6 (ZBTB4 and ZBTB10) and several more for which antibodies either gave ambiguous results or were unavailable (BEND3, ETV6, MBD1, ZBTB12 and ZBTB20, all of which are amongst those with extreme H/L ratios; Fig 3). ARID3A was included in this set because preliminary analysis of the data of the experiment of S2 Fig identified it as a protein in the purified fraction that was sensitive to HSV-1 infection, consistent with published studies [43]. Although the H/L ratio of ARID3A was not significantly reduced in the experiment of Fig 2, it was identified as a likely sumoylated substrate (S1 Table, sheet 2) and a related protein (ARID4A) was reduced during infection (Fig 3). NACC2 was included in the analysis because it was the highest scoring protein ID of the experiment of Fig 2 that did not achieve the cut-off values of H/L ratio change in the triple labeled experiment. All the proteins were expressed in the inducible system (most by 2 h after induction), and most gave a major band close to the predicted molecular weight plus minor slower migrating species that are likely sumoylated products (S4 Fig). ZBTB4 was the least efficiently expressed of these proteins, and any sumoylated forms were below the level of detection in the presented exposure (but see Fig 8, in which putative sumoylated forms are visible).

The various cell lines were then treated with doxycycline for the appropriate length of time, the doxycycline was then washed out and the cells infected at moi 10 for 8 h with either wt or ICP0 null mutant HSV-1 (lanes marked dl). The extracts were also analyzed for the efficiency of viral gene expression in each instance (Fig 8). Where visible on the blot exposures presented, the slower migrating probable sumoylated forms were invariably lost during wt but not ICP0 null mutant infection, indicating that their loss is, directly or indirectly, dependent on ICP0. The major likely unmodified forms of ZBTB4, ZBTB10, ZBTB12, MBD1, BEND3 and NACC2 were also reduced to a greater or lesser extent in the wt virus infected cells (Fig 8). In contrast, potential sumoylated forms of ZBTB4, ZBTB10 and ETV6 appeared to increase in abundance in the ICP0-null mutant infected samples, which may be related to the overall accumulation of sumoylated species that occurs during the mutant virus infection [18,19]. The reduction in the major form for ZBTB4 observed here is consistent with the data of Fig 6, with both experiments being conducted in HepaRG-based cells, and in contrast to the infection time course of Fig 7 (performed in HF cells), supporting the possibility of cell type differentials in the behaviour of certain proteins during HSV-1 infection.

Taking into account all the proteins analyzed in Figs 5, 6 and 8, of the 124 proteins listed in Fig 5, five had been defined previously as decreasing in abundance during HSV-1 infection (although not all have well characterized sumoylated forms). We have also analyzed 11 further proteins that had not been studied in HSV-1 infection, finding that all showed evidence of sumoylated forms which were sensitive to HSV-1 infection, and in some cases their likely unmodified forms also. Thus of the 13% of the proteins in Fig 5 investigated, 100% were confirmed as behaving as predicted from the proteomic analysis. This very high confirmation rate gives much confidence about the overall validity of the proteomic analysis.

### Mechanisms of degradation of cellular proteins during HSV-1 infection

There are several mechanisms that could reduce the amounts of the candidate proteins identified in this study. The prime aim of the project was to identify sumoylated forms of proteins that are degraded in an ICP0-dependent manner, and it is likely that these constitute a major grouping. However, proteins that alter in abundance during HSV-1 infection that are either unsumoylated or modified to only a very minor degree may also be detected by this methodology, allowing the possible identification of substrates of ICP0 that are degraded in a SUMO-independent manner. Host cell proteins may also become less abundant during HSV-1 infection in an ICP0-independent manner, either through induced degradation as a consequence of virus infection in general or more passively because of reduced rates of host transcription. Distinguishing between substrates that are degraded by ICP0-dependent and-independent mechanisms during HSV-1 infection is not always straightforward due to the low infectivity of ICP0-null mutant HSV-1. For example, initial studies indicated that IFI16 was degraded by ICP0 during HSV-1 infection [32], but it later emerged that the degradation could occur in the absence of ICP0 in conditions in which the mutant infection was progressing as rapidly as the wt [31]. Therefore we have investigated the degradation of selected proteins of interest in cells induced to express ICP0 in the absence of infection (as described in [42]).

**Fig 9:**
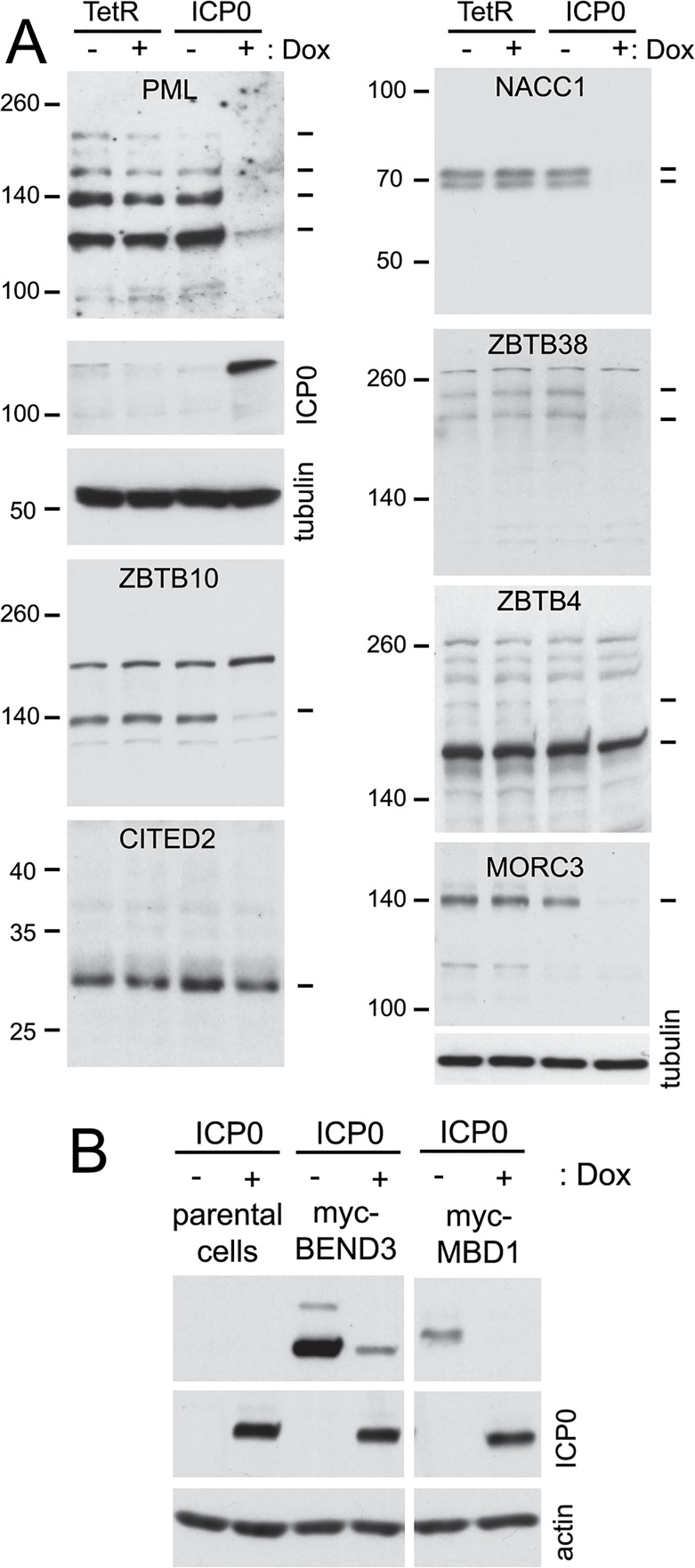
Degradation of selected cellular proteins by ICP0 in the absence of infection. A. HA-TetR and HA-cICP0 cells were treated with doxycycline (100 ng/ml) for 24 h (+) or left untreated (-), then whole cell extracts were prepared and analyzed by western blotting for the indicated proteins using antibodies directed against the endogenous proteins. The dashes on the right indicate the major presumed specific bands (determined on the basis of analyses in previous figures) in each case. Except in the cases of CITED2 and ZBTB4, these major bands decrease in the presence of ICP0. The upper dash in the ZBTB4 panel indicates a potential sumoylated form that is sensitive to ICP0. (B). Analysis of HA-cICP0 cells (left, lanes 1 and 2) and derivatives of HA-cICP0 cells that had been transduced with lentivirus vectors expressing myc-tagged BEND3 (left, lanes 3 and 4) and MBD1 (right), before and after induction of ICP0 expression, illustrating that ICP0 is sufficient to degrade BEND3 and MBD1.

Control HA-TetR and ICP0 inducible cells were treated or not with doxycycline then the whole cell extracts were analyzed by western blotting. As reported previously [42], PML is efficiently degraded in this system (Fig 9, top left). The stability of the endogenous forms of proteins MORC3, ZBTB10, ZBTB38, NACC1, ZBTB4 and CITED2 after induction of ICP0 expression was analyzed because of the availability of antibodies that detect endogenous levels of the proteins. It is clear that the first four of these proteins can be degraded by ICP0 in the absence of infection, thereby identifying a number of previously undescribed ICP0 substrates. CITED2, on the other hand, appears only be degraded in HSV-1 infected cells, while the major form of ZBTB4 (but not a more slowly migrating, potential sumoylated form of the protein; see also Fig 7) appears to be relatively stable in the presence of ICP0. The relative loss of the major form of ZBTB4 during infection seen in Figs 5 and 8 does not seem to occur in the presence of ICP0 but the absence of infection, even though the protein was stable during infection with an ICP0-null mutant virus.

To expand the repertoire of proteins for which we could test the effect of ICP0 specifically we constructed vectors that expressed blasticidin resistance and myc tagged versions of BEND3 and MBD1 constitutively. ICP0 inducible cells were transduced with these vectors, then induction of ICP0 expression revealed that both proteins could be degraded by ICP0 alone (Fig 9B).

### Influence of sumoylation on the degradation of selected cellular proteins

**Fig 10:**
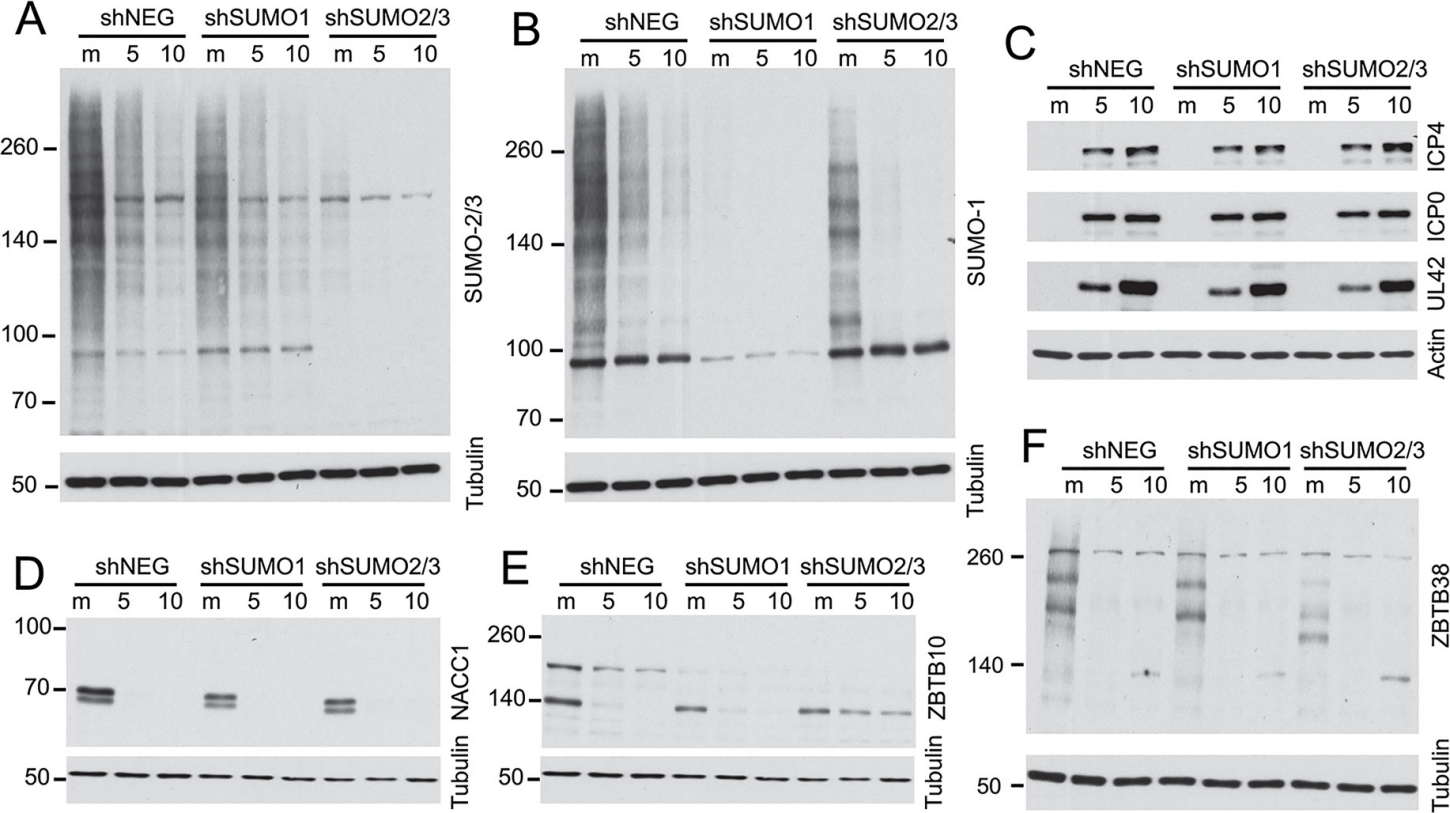
Impact of depletion of SUMO1 or SUMO2/3 on degradation of selected proteins during HSV-1 infection. HepaRG cells depleted of SUMO1 or SUMO2/3 simultaneously were prepared by lentiviral transduction (see Methods) then analyzed by western blotting for the abundance of SUMO2/3 (A) or SUMO1 (B) conjugates after infection with wt HSV-1 (MOI 10) for 5 or 10 h in comparison with control cells (shNEG). The same samples were analyzed for the efficiency of viral gene expression (C) and the abundance of NACC1 (D), ZBTB10 (E) and ZBTB38 (F).

We investigated whether the degradation of selected proteins was influenced by the presence of SUMO2/3 by analyzing cells transduced by a lentivirus expressing multiple shRNAs that target both SUMO2 and SUMO3. These cells were highly depleted of SUMO2/3 (Fig 10A) and they exhibited highly reduced levels of sumoylated PML, disrupted PML NBs and increased replication of ICP0-null mutant HSV-1 (M. Glass, submitted for publication). In parallel, cells depleted of SUMO1 were also examined (Fig 10B). Depletion of the SUMO isoforms did not affect the efficiency of wt HSV-1 gene expression during high multiplicity infections (Fig 10C) and the remaining SUMO1 and SUMO2/3 proteins in these cells were sensitive to HSV-1 mediated reductions (Fig 10A and 10B). We analyzed examples of proteins for which antibodies that detect the endogenous proteins were available, finding that degradation of NACC1 was unaffected by depletion of SUMO2/3 (Fig 10D), while ZBTB10 was more stable in the SUMO2/3 depleted cells (but not the SUMO1-depleted cells) (Fig 10E). These results indicate that degradation of ZBTB10 is ICP0-dependent and is influenced by the abundance of SUMO2/3. ZBTB38 gave a marked interesting result, in that the bands detected by the antibody were clearly shifted in mobility in the SUMO2/3 depleted cells, but not in the SUMO1 depleted cells, and the novel ZBTB38 bands were lost in the infected cells (Fig 10F). These data indicate that ZBTB38 may be preferentially modified by SUMO2/3 compared to SUMO1, and its HSV-1 induced degradation is dependent on ICP0 but not on modification by SUMO2/3.

### Influence of the SUMO2 interaction motif in ICP0 mediated degradation

**Fig 11:**
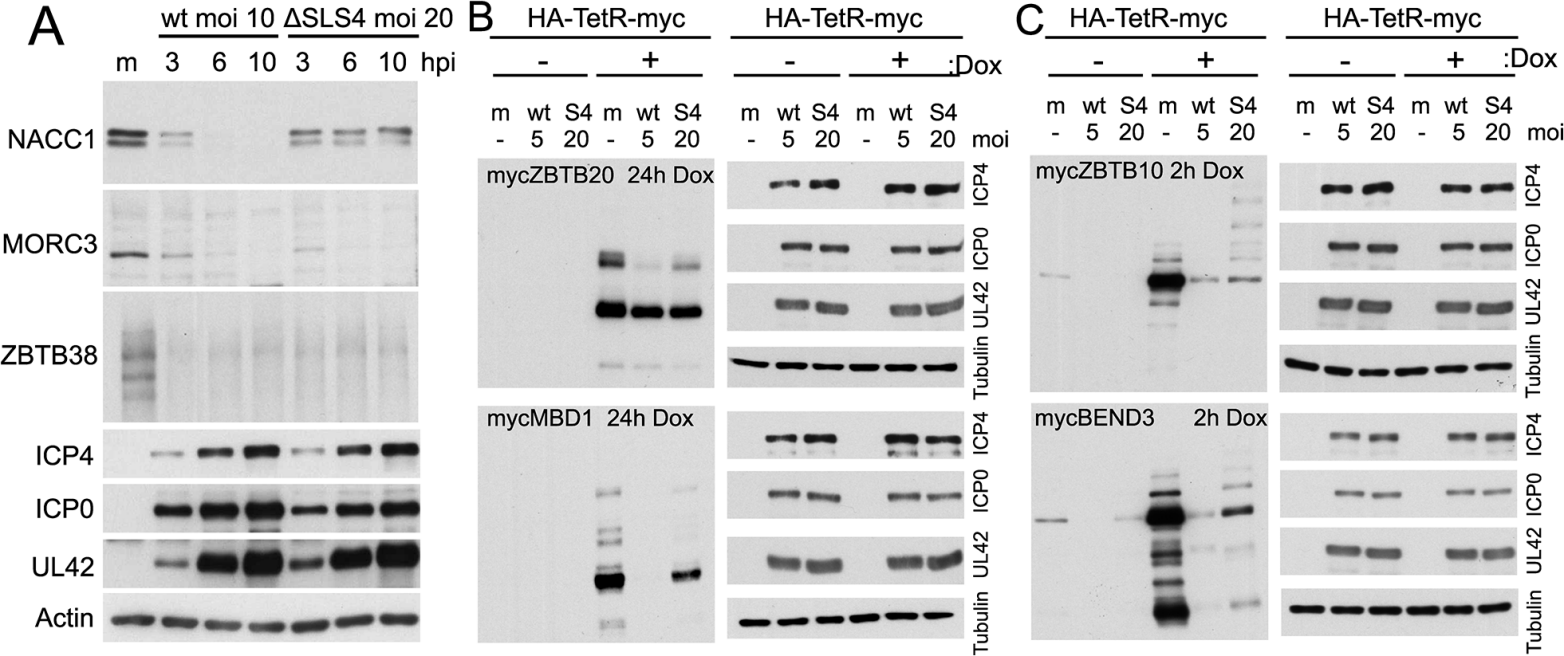
Influence of a SUMO interaction motif within ICP0 on the stability of selected proteins during HSV-1 infection. A. HepaRG cells were infected with wt or mSLS4 mutant HSV-1 and samples taken at 3, 6 and 10 h after infection were analyzed for the abundance of NACC1, MORC3 and ZBTB38 as indicated. The MOI were adjusted to ensure similar levels of viral gene expression (10 for wt, 20 for mSLS4), as shown in the ICP0, ICP4 and UL42 panels. B and C. HepaRG cells transduced to express ZBTB20, MBD1 (B) and ZBTB10 and BEND3 (C) in an inducible manner were left uninduced or treated with doxycycline for the indicated times to induce expression of the myc tagged proteins. These cells were then infected with wt or mSLS4 mutant HSV-1 for 8 h (MOI 5 and 20 respectively), to ensure similar levels of viral protein expression (as indicated by the panels on the right) then analyzed for the relevant proteins by detection of the myc tag.

ICP0 includes several candidate SUMO interactions motifs (SIMs), one of which was shown by yeast 2 hybrid assay to bind to SUMO2, and was hence termed SIM-like sequence (SLS) -4 [18]. A recombinant HSV-1 mutant (mSLS4) was constructed with mutations in SLS4, which, although causing only a slight defect in itself, resulted in a highly defective phenotype when present in conjunction with mutations in other SLS motifs [44]. Mutant SLS4 was also found to be defective in degrading sumoylated forms of PML isoforms other than PML.I (when these were expressed in isolation), although it retained the ability to degrade PML.I in a sumoylation-independent manner [44]. As PML.I is the most abundant PML isoform and it interacts with all the others, mSLS4 retains the ability to degrade endogenous PML isoforms. We therefore investigated whether ICP0 lacking functional SLS4 could degrade a selection of the proteins of interest during infection either of normal cells or of transduced cell lines expressing a myc-tagged version of selected proteins, depending on antibody availability. The results indicated that the degree of degradation of a given protein could be influenced by the SLS4 mutation in ICP0 when expressed in the context of infection. For example, endogenous NACC1 appeared more stable during mSLS4 than wt virus infection whereas MORC3 was equally sensitive to the two viruses (Fig 11). We present three examples from the myc tagged protein data, including ZBTB20 (for which the sumoylated form is more resistant to loss in mSLS4 than wt virus infection, while the major band is relatively unchanged), MBD1 (for which all forms are reduced to a lesser extent in the mSLS4 compared to wt infection), and ZBTB10 (for which the major band is equally sensitive in the two infections while the abundance of likely sumoylated species increases) (Fig 11). The reasons for this last observation will be considered in the Discussion, but note that the sumoylated forms of tagged ZBTB10 also increased in abundance in cells infected with ICP0-null mutant HSV-1 (Fig 9).

Taken together, the results of Figs 9 to 11 indicate a range of factors which influence the reduction in abundance of these proteins during HSV-1 infection. These include via mechanisms for which ICP0 is insufficient (such as CITED2), or ICP0-dependent mechanisms which can be influenced or not by the abundance of SUMO2/3 (ZBTB10 or NACC1 respectively) and/or by the presence of the SUMO2 interaction motif of ICP0. This analysis illustrates the complexities that regulate cellular protein stability during HSV-1 infection, and each example requires careful analysis to determine all the factors involved; there is no one single simple mechanism at play.

## Discussion

This study is the first to report a comprehensive analysis of the cellular proteins whose abundance is affected by HSV-1 infection, with specific focus on proteins modified by SUMO2. The loss of SUMO-modified PML and Sp100 in an ICP0-dependent manner during HSV-1 infection has been reported previously [18,19,40,41,45], and these proteins play important roles in an intrinsic immune response to HSV-1 infection [9,10]. The bulk of SUMO-conjugated proteins are also reduced following infection with wt HSV-1 but increased during ICP0 null mutant infection [18,19]. The biological relevance of these observations was supported by the finding that disruption of the SUMO pathway through knockdown of Ubc9 enhanced the replication of an ICP0-null mutant HSV-1 [18]. These results prompted the question as to the identity of the affected SUMO-modified proteins and their role in the context of HSV-1 infection. To address these questions we used Mass Spectrometry (MS)-based quantitative proteomics analysis of HepaRG cells expressing His-tagged SUMO2 (HA-HisSUMO2 cells) with and without HSV-1 infection. The use of SILAC Light, Heavy and Medium media for HSV-1 infected and uninfected HA-HisSUMO2 cells and uninfected control HA-His only cells, respectively, allowed for the relative fold changes to be calculated for His-SUMO2 purified proteins and unmodified proteins following infection. This analysis identified with high confidence 877 cellular sumoylated proteins under these experimental conditions, of which 521 (59%) were in common with a compilation of the largest previous SUMO substrate identification proteomics studies [23–28]. Following HSV-1 infection 260 of these proteins changed in abundance with SigB values of less than 0.1, indicating some specificity in the targeted group of proteins; relative loss of sumoylated species is not simply a consequence of sumoylation per se, but the identity of the sumoylated species is also important. This is further illustrated by the fact that only 14% of these proteins decreased in abundance by over 3-fold and only 1% by 7-fold or more. Our analysis of specific proteins in this group has therefore been restricted to a subset of those most highly affected by HSV-1 infection.

Identification in our MS dataset of the sumoylated forms of PML and Sp100 as proteins whose abundance is significantly reduced during infection supported the validity of the experiment, as did the lack of change in sumoylated RanGAP1, which is not affected by HSV-1 [19]. Of the other proteins whose sumoylated forms reduced by a factor of at least 3-fold, a significant proportion may be linked to transcriptional regulation or chromatin related pathways (Fig 5). This supports the view that one of the most recognized functions of sumoylation is to regulate transcription [46–49]. SUMO-modification of transcriptional regulators is often described as having an inhibitory effect on transcription [47,50–52], however, there have been instances where SUMO-modification enhances transcription factor activity [53]. SUMO-modification of proteins in the context of transcriptional regulation may include transcription factors themselves, transcriptional co-regulators, and chromatin-remodeling proteins [46]. Sumoylation of these proteins may regulate their DNA binding activity, subcellular localization, assembly of multi-component complexes, interaction between transcription factors and co-regulators, and also DNA repair pathways and chromatin structure [46,54,55]. The reduction in the levels of the sumoylated forms of the number of proteins linked to these functions, and in some cases also their total abundance, would likely in normal circumstances have profound effects on the cell. In the samples analyzed here, the cells are subject to an extremely active and soon to be fatal infection, so even drastic changes to cellular gene expression may be inconsequential. The more interesting questions concern the consequences these changes may have on viral gene expression, and the potential identification of previously unrecognized preferential substrates for ICP0-mediated degradation. Therefore our analysis was driven first by authentication of a number of potentially interesting proteins, then by investigation of the role of ICP0 in their desumoylation or degradation.

We verified several example proteins on the list of sumoylation substrates that change during HSV-1 infection through a combination of western blotting of total protein extracts and His-SUMO2 purified proteins from mock and infected HA-HisSUMO2 cells (Fig 6), time course of degradation of endogenous proteins in whole infected cell extracts (Fig 7), and analysis of epitope tagged protein expression in stable cell lines (Fig 8). Evidence in favor of the sumoylation of these proteins, and the loss of these sumoylated forms (and in some cases their unmodified species) was obtained in one or more of these approaches. Overall, 13% of the proteins listed in Fig 5 were subjected to verification, with entirely positive results.

The loss of bulk sumoylated species following HSV-1 infection is more pronounced in human fibroblasts than HepaRG cells [18], and therefore we also analyzed the fate of several designated sumoylated proteins (ZBTB4, ZBTB10, ZBTB38, NACC1, and MORC3) over a time course of HSV-1 infection of HFs (Fig 7). All of these proteins decreased in abundance following wt HSV-1 infection, some as early as 3 h p.i. This suggests many proteins may be targeted much earlier than the 12 h time point used for the MS experiments (chosen to ensure complete infection and maximal effects). The available antibodies to these endogenous proteins do not always recognize a clearly sumoylated form, but it is clear that in most cases even the major unmodified form is sensitive to HSV-1 infection. Similar results were obtained in HepaRG cells, and these proteins remained stable during infection with ICP0-null mutant HSV-1.

In some cases, problems with lack of availability, specificity, or affinity of available antibodies were overcome using an inducible lentiviral expression system, in which the level of protein expression can be regulated by time of induction. In all cases, likely sumoylated bands were detected (S4 Fig) and these, together in some cases with the unmodified forms, were sensitive to HSV-1 infection (Fig 8). These proteins were however stable during infection with an ICP0 null mutant virus used at a multiplicity allowing an equivalent level of infection between wt and ICP0 null virus infected cells (Fig 8). To investigate whether ICP0 alone in the absence of infection is sufficient to decrease putative SUMO-modified protein bands, we utilized cells that can be induced to express ICP0 at levels equivalent to those during wt infection [42]. These experiments illustrated that ZBTB4, ZBTB10, ZBTB38, NACC1, MORC3, BEND3 and MBD1 all suffered a loss of total protein or likely sumoylated bands in the presence of ICP0, illustrating that ICP0 can cause this phenotype in the absence of other viral proteins. It is likely that the reduced abundance of many other proteins identified in this study is also ICP0-dependent. Whether ICP0 induces desumoylation or degradation of the sumoylated forms of these proteins requires further investigation, but in those instances where there is a loss of total protein suggests that the sumoylated species are being degraded rather than desumoylated.

We investigated the role of the sumoylation itself in substrate targeting in a limited number of instances through two approaches, firstly using SUMO depleted cells, and secondly analysis of the effects of a mutant form of ICP0 with a SUMO2 interaction motif inactivated. A complete analysis of these issues is beyond the scope of this paper, but the results demonstrate that sumoylation can play a role in the response of a protein to HSV-1 infection, but that the details may differ between proteins. It is intriguing that infection with the mSLS4 virus causes an increase in overall SUMO conjugate levels [44], and this is reflected in either reduced loss of presumed sumoylated forms of certain proteins during mSLS4 infection, or indeed an increase in their abundance (as in the case of ZBTB10; Fig 11). Presumed sumoylated forms of ZBTB10 and ZBTB4 also increase during ICP0 null mutant HSV-1 infection (Fig 8). These results confirm the role of sumoylation and SUMO-SIM interactions in some of the effects we observe. It is more difficult to determine whether these functions also impact on the degradation of the unmodified forms of selected proteins, as ICP0 can also target proteins in a sumoylation-independent manner [44], and it also includes other potential SIMs. We did not analyse the activity of a form of ICP0 that lacks multiple potential SIMs in this study because this mutant is highly defective in degrading all substrates previously analyzed [44].

Proteins containing a zinc finger (ZF) and/or BTB (broad-complex, tramtrack and bric-à-brac) domain were prominent on the list of those showing a greater than 3-fold reduction in the purified fraction following wt HSV-1 infection (Fig 5). These ZF and BTB domains are likely to bind DNA and mediate protein:protein interactions, respectively. Apart from being predicted to play roles in transcriptional regulation, the precise functions for most ZBTB proteins, the proteins they interact with and the genes they regulate are yet to be discovered [56]. Thus loss of sumoylation of ZBTB proteins may control transcription of cellular genes required for a successful immune response to the infection, or prevent repression of transcription of viral genes. In addition to potential roles in chromatin and transcription-related pathways, many BTB proteins are now known to act as substrate-specific adaptors for Cullin3-based E3 ligases [57] and hence play a role in modulating protein stability. Some of the known functions of the BTB and ZBTB and other proteins validated in this study are summarized in Table 1.

**Table 1:** Brief information on sumoylated proteins validated in this study.

Protein	Evidence for sumoylation	Reported functions	References
ZBTB4	Yes [24]	Binds methylated DNA; Transcriptional repressor; associates with Sin3 co-repressor	[83–85]
ZBTB10	Yes [24,26]	Binds methylated DNA; Transcriptional repressor	[86,87]
ZBTB12	Yes [26]	Associated with polymorphisms in ER positive breast cancers	[88]
ZBTB20	Yes [28,89]	Related to BCL-6 and PLZF; transcriptional repressor; inhibits IκB transcription	[90,91]
ZBTB38	Yes [24,89]	Binds methylated DNA; transcriptional repressor; binds co-repressors	[83,92]
NACC1	Yes [26]	BTB/POZ domain protein; interacts with CoREST, HDAC3, HDAC4, cullins; expression levels linked with several carcinomas	[93–97]
NACC2	This paper	BTB and BEN domain protein; interacts with NuRD chromatin remodelling complex; inhibits HDM2 transcription	[98]
BEND3	Yes [58]	BEN domain protein; transcriptional repressor; interacts with chromatin remodelling complexes; causes chromatin condensation	[58,99]
ARID3A	Yes [100]	Transcriptional regulator; interacts with Ubc9, Sp100, ICP0; desumoylates PML and disrupts PML NBs; degraded by ICP0	[43,101–103]
ETV6	Yes [26,65]	ETS family transcription factor; transcriptional repressor; interacts with co-repressor complexes	[65,67–70]
MORC3	Yes [26,60]	Zinc finger protein; localizes to PML NBs; influences Sp100 and p53 localization to PML NBs; sumoylated form interacts with PML.I; autoantigen in dermatomyocitis	[59,60,104]
MBD1	Yes [26,49]	Methyl-CpG binding protein; also binds unmethylated DNA and represses transcription; interacts with HDACs and SETDB1	[61–64]

In addition to the ZBTB and BTB proteins listed in Table 1, we also validated some other proteins implicated in chromatin-related pathways and transcriptional repression, such as BEND3, MORC3, MBD1 and ETV6. It is intriguing that BEND3 promotes heterochromatinization and that sumoylation is important for its repressive activities [58], while MORC3 is a PML NB component that interacts with PML in a SUMO-dependent manner [59,60]. MBD1 is the largest of the methyl-CpG binding domain (MBD) family of proteins [61] that can also bind to unmethlyated DNA to mediate transcriptional repression [62,63] via its interaction with HDACs [63,64]. ETV6 is also a transcriptional repressor that can be sumoylated [65,66] and which interacts with transcriptional co-repressors and histone deacetylases [67–71].

While the biological significance of the changes in abundance and/or sumoylation status of these and the other proteins listed in Figs 3 and 5 remains to be determined, we note that of the 124 proteins whose sumoylation status changes by 3-fold or more during HSV-1 infection (Fig 5), four (PML, Sp100, ATRX and IFI16) have already been reported to affect the efficiency of ICP0 null mutant HSV-1 infection [9,10,12,31,72]. Given the number of proteins listed in Fig 5 that have potential roles in gene expression and chromatin-related pathways, it seems likely that future studies will reveal more such examples.

In addition to cellular proteins, we also identified a number of viral proteins that had higher than predicted molecular weights in the His-SUMO2 purified fraction. Excluding glycoproteins, these included nuclear protein UL3, capsid portal protein UL6, protein kinase US3, deoxyribonuclease UL12, tegument protein US11, DNA polymerase processivity subunit UL42, transcriptional regulator ICP4, ICP0, and regulatory protein ICP22 (Fig 4). There is no previous report of HSV-1 proteins being modified by SUMO, although the UL42 homologues expressed by HCMV (UL44) [37], and possibly that of EBV (BMRF1) [73] can be sumoylated. Thus the potential to be sumoylated may be a feature of the DNA polymerase accessory subunits expressed by herpesviruses. Of the HSV-1 proteins listed above, US3, UL12 and UL42 contain potential SUMO conjugation sites and analysis of His-SUMO2 purified proteins with available antibodies revealed likely sumoylated species, albeit in low amounts, for UL6, UL12, ICP0 and UL42. Further analysis will be required to investigate the extent and consequences of this potential sumoylation. In general, there are several reports of viral proteins subject to sumoylation, and in some cases this is an important aspect of their activity (reviewed in [74,75]).

In summary, post-translational modification of proteins with SUMO has important implications for protein function, and has roles in many pathways of importance for viral infections, including transcriptional regulation, and innate and intrinsic immune responses (reviewed in [74,75]). This proteomics study has provided a large data set opening many avenues of research for not only herpes virology, but also other areas where the sumoylation of proteins is of heightened interest. The validation using independent methods of the changes in abundance of many proteins identified by the MS approach lends considerable confidence to the overall utility of this study, and we have documented several previously unrecognized examples of proteins that are subject to ICP0 mediated degradation. In that a number of known biologically relevant substrates of ICP0 were identified in our study, it is likely that future studies on the basis of this analysis will reveal important novel aspects of the regulation of herpesvirus infection.

## Materials and Methods

### Cells

Human diploid foreskin fibroblasts (HFs, obtained from Dr Thomas Stamminger, University of Erlangen), HEK-293T human embryo kidney cells (American Type Culture Collection CRL-11268) and human osteosarcoma cells (U2OS, American Type Culture Collection HTB96) cells were grown in Dulbecco’s Modified Eagles’ Medium (DMEM) supplemented with 10% fetal calf serum (FCS). Baby hamster kidney cells (BHK-21, obtained from original Glasgow MRC Virology Unit stocks) were grown in Glasgow Modified Eagles’ Medium (GMEM) supplemented with 10% new born calf serum and 10% tryptose phosphate broth. HepaRG cells [76] were grown in William’s Medium E supplemented with 10% fetal bovine serum Gold (PAA Laboratories Ltd), 2 mM glutamine, 5 μg/ml insulin and 0.5 μM hydrocortisone. Derivatives of HepaRG cells expressing the tetracycline repressor (HA-TetR cells) and wt ICP0 in a doxycycline inducible manner (HA-cICP0 cells) have been described previously [18,42]. HepaRG cells transduced with lentiviruses expressing multiple anti-SUMO1 or a combination of anti-SUMO2 and antiSUMO3 shRNAs have been described elsewhere (M. Glass, manuscript submitted), as has the control lentivirus expressing an shRNA that does not target any human gene (shNeg) [11]. All cell growth media were supplemented with 100 units/ml penicillin and 100 μg/ml streptomycin. Lentivirus transduced cells were maintained with continuous antibiotic selection, as appropriate.

### Viruses

HSV-1 wild type (wt) strain 17 and mutant *dl*1403 [77] were the wt and ICP0-null mutant strains used. Virus mSLS4, which expresses a form of ICP0 with an inactivated SIM-like sequence SLS4, has been described previously [44]. These viruses were grown in BHK cells and titrated in U2OS cells, using 1% human serum in the overlay. ICP0 is not required for HSV-1 plaque formation in U2OS cells [78], therefore allowing a true comparison of the titres of wt and mutant virus stocks. Estimation of plaque formation efficiencies in cell lines expressing SUMO family members was performed using wt and ICP0 null mutant HSV-1 isolates (viruses *in*1863 and *dl*1403CMV*lacZ* respectively) that express a β-galactosidase marker gene linked to the HCMV promoter, as described previously [42]. Briefly, cells were seeded into 24-well dishes then infected with 3-fold serial dilutions of the viruses the following day. After 24 h incubation in the presence of additional 1% human serum, the cells were stained for β-galactosidase activity as described [42]. Relative plaque forming efficiencies were calculated by determining the number of plaques in each cell line at a given dilution of virus, then calculating fold changes in plaque number compared to controls cells at the same dilution. Averages and standard deviations were calculated from at least three independent determinations.

### Lentiviral vector plasmids and transductions

Lentivirus vector plasmid pLVX-6His-SUMO2, in which the human SUMO2 cDNA with a polyhistidine tag was inserted between the XhoI and XbaI sites of pLVX-IRES-Puro (Clontech), and pLVX-6His (a control with only the tag sequence) were kindly provided by Ben Hale. Plasmids with cDNAs of selected cellular proteins were either purchased from Source Bioscience or were gifts from Pierre-Antoine Defossez (MBD1 transcript variant 3, ZBTB4). The cDNAs were amplified by PCR using primers containing suitable restriction sites and encoding an in frame N-terminal myc tag, then the products were inserted in place of the ICP0 cDNA in doxycycline inducible lentiviral vector pLKO.DCMV.TetO-cICP0 (pLDT-cICP0) [42]. Lentiviral vectors expressing myc tagged versions of BEND3 and MBD1 in a constitutive manner were constructed by inserting the relevant cDNAs in place of the EYFP-PML cDNA in plasmid pLKOneo.gD.EYFP-PML.I [79] which had been modified by inserting the blasticidin resistance coding region in place of that for neomycin. Lentivirus transductions of were performed as described [9], with stable cell lines selected using puromycin (1 μg/ml, reduced to 0.5μg/ml for subsequent passage), G418 (0.5 mg/ml) or blasticidin (1 μg/ml), or combinations thereof, as relevant.

### Cell culture, SILAC labeling and virus infection

HA-HisSUMO2 and HA-His Only cells were cultured in SILAC DMEM lacking L-lysine and L-arginine, which were replaced with normal (light; L), heavy (H) or medium (M) stable isotopically labeled forms of these amino acids (all SILAC medium reagents were sourced from Cambridge Isotope Laboratories). These media were supplemented with 10% dialyzed fetal bovine serum (FBS), 100 units/ml penicillin and 100 μg/ml streptomycin and 0.5 μg/ml puromycin. Mock infected HA-HisSUMO2 cells were cultured in H medium (^13^C_6_^15^N_2_-lysine, Lys^8^, and ^13^C_6_^15^N_4_-arginine, Arg^10^), wt HSV-1 infected HA-HisSUMO2 cells were cultured in L medium (isotopically normal; Lys^0^, Arg^0^), and mock infected HA-His Only cells were cultured in M medium (4,4,5,5-D^4^-lysine, Lys^4^, and ^13^C_6_-arginine, Arg^6^). Cells were cultured for six population doublings in their respective SILAC media before being expanded into 12 x 150 mm dishes for each condition. HA-HisSUMO2 cells cultured in SILAC Light medium were infected at an MOI of 10 plaque forming units per cells for 12 h.

### Nickel affinity purification

The method used was essentially as described [27]. Cells were washed twice with phosphate buffered saline (PBS), then lyzed in denaturing nickel sample buffer [6 M guanidinium hydrochloride (Merck), 94.7 mM Na_2_HPO_4_ (VWR Prolabo), 5.3 mM NaH_2_PO_4_ (VWR Prolabo), 10 mM Tris/HCl (Roche) pH 8.0, 20 mM imidazole (Sigma), 5 mM β-mercaptoethanol (Sigma), complete EDTA free protease inhibitor cocktail (Roche)], and stored at -70°C. Once thawed, equal amounts of protein from each Light, Medium and Heavy SILAC treatment (determined by BCA assay (Pierce) and confirmed by Silver staining after SDS-PAGE) were mixed and sonicated. Sonicated lysates were then centrifuged at 1,000 x g at 4°C for 10 min followed by passage through a 0.45 μm filter. Lysates were then added to 50 μl Ni^2+^ NTA agarose beads (Qiagen), pre-equilibrated with denaturing nickel sample buffer, and incubated with rotation at 4°C for 24 h. Beads were centrifuged out of suspension 1,000 x g at 4°C for 10 min. Beads were washed by centrifugation at 720 x g for 2 min in 1 ml buffer in a Lo-Bind Eppendorf tube as follows: once in denaturing nickel sample buffer, twice with wash buffer pH 8.0 [8 M urea (Sigma), 94.7 mM Na_2_HPO_4_, 5.3 mM NaH_2_PO_4_, 10 mM Tris/HCl pH 8.0, 20 mM imidazole, 5 mM β-mercaptoethanol, complete EDTA free protease inhibitor cocktail], twice in wash buffer with the pH reduced to 6.3, and once with a final wash in a new Lo-Bind Eppendorf tube in wash buffer pH 8.0. Bound proteins were then eluted from beads in 40 μl nickel resin elution buffer [2x LDS (Invitrogen), 1x reducing reagent (Invitrogen), 200 mM imidazole] at room temperature with agitation for 10 min, followed by boiling for 2 min. Samples were stored at -20°C.

### Proteomic analysis

For both quantitative proteomic experiments a ‘crude’ sample was prepared by TCA precipitation of proteins from a sample of the mixed lysates prior to nickel affinity purification; 35 μl of the protein mixture in the 6 M guanidine hydrochloride buffer (see above), containing about 70 μg of protein, was mixed with 400 μl 10% trichloroacetic acid (TCA), incubated in ice for 20 min and centrifuged at 19000 x g for 15 min at 4°C. The pellet was washed with 1 ml 100% ethanol at 4°C and re-centrifuged at 19000 x g for 15 min at 4°C. Supernatants were aspirated and the pellets dried in a gyrovap before resuspension in 80 μl 1.5 x LDS sample buffer containing reducing agent (Invitrogen). Then 35 μl of this and the nickel affinity chromatography elutions (representing between 20 and 40 μg of total protein) were fractionated by polyacrylamide gel electrophoresis containing SDS (NuPage 10% polyacrylamide, Bis-Tris with MOPS buffer—Invitrogen) and stained with Coomassie blue. For each experiment both crude and pure lanes were excised into identical slices according to apparent MW of markers, as indicated in Figs 2 and 3. Peptides were extracted from each slice by tryptic digestion [80], including alkylation with chloroacetamide.

Peptide samples were analyzed by LC-MS/MS on a Q Exactive mass spectrometer (Thermo Scientific) coupled to an EASY-nLC 1000 liquid chromatography system via an EASY-Spray ion source (Thermo Scientific) running at 75 μm x 500 mm EASY-Spray column. Elution gradient durations of 150 min and 240 min were used. Data were acquired in the data-dependent mode. Full scan spectra (*m/z* 300–1800) were acquired with resolution *R* = 70,000 at *m/z* 400 (after accumulation to a target value of 1,000,000 with maximum injection time of 20 ms). The 10 most intense ions were fragmented by HCD and measured with a target value of 500,000, maximum injection time of 60 ms and intensity threshold of 1.7e3. A 40 second dynamic exclusion list was applied.

Raw MS data files were processed together with the quantitative MS processing software MaxQuant (version 1.3.0.5) [30,81] Enzyme specificity was set to trypsin-P as required. Cysteine carbamidomethylation was selected as a fixed modification and methionine oxidation, protein N-acetylation and gly-gly adducts to lysine were chosen as variable modifications. The data were searched against a target/decoy human database in addition to the HSV-1 database (GenBank accession number JN555585.1). Initial maximum allowed mass deviation was set to 20 parts per million (ppm) for peptide masses and 0.5 Da for MS/MS peaks. The minimum peptide length was set to 7 amino acids and a maximum of four missed cleavages. 1% false discovery rate (FDR) was required at both the protein and peptide level. The ‘match between runs’ option was selected with a time window of two minutes. Data were output twice; firstly separated by ‘crude’ and ‘pure’ conditions, and secondly such that each digestion of each gel slice was considered a single ‘experiment’. The former is used for overall protein ratio changes, and the latter for apparent MW analysis [29].

### Definition of putative SUMO2 substrates from proteomics experiments

In the triple SILAC labeled experiment one condition represented non-infected cells expressing the 6His sequence only, (HA-His Only cells) (See Fig 2). This was in the isotopically ‘medium’ condition (M), and so any ratio comparing this condition with the ‘light’ (L) or ‘heavy’ (H) conditions where 6His-SUMO-2 was expressed, i.e. M/L and H/M can be used as comparison between HA-HisSUMO2 and HA-His Only purifications. As SUMO2 substrates by definition should be more abundant in H or L conditions than M, substrates will be characterized by large H/M and small M/L ratios. For log_2_ M/L and log_2_ H/M ratios, two cutoffs of <-1.790 and >0.822 respectively were used. If applied to pure ratios this defined 877 putative SUMO2 substrates, representing 30.8% of all identifications. The same criteria applied to the crude ratios shortlisted 36 proteins, representing 0.65% of all identifications. By this method the false discovery rate for SUMO2 substrate definition is estimated to be below 1%.

### Western blots and antibodies

For analysis of whole cell extracts, cells were seeded into 24-well dishes at 1 x 10^5^ cells per well, then infected or treated with doxycycline the following day, as described in the figure legends. Cell monolayers were washed twice with PBS before harvesting in SDS-PAGE loading buffer. Proteins were resolved on 7.5% SDS-polyacrylamide gels, then transferred to nitrocellulose membranes by western blotting. Antibodies directed against the following proteins were used: 6xHis monoclonal antibody (mAb) (ab18184, Abcam), SUMO2/3 rabbit polyclonal antibody (rAb ab3742, Abcam), RanGAP1 (mAb 33–0800, Invitrogen), PML 5E10 mAb [82], Sp100 rAb SpGH [5], actin (mAb AC-40, Sigma), myc tag (mAb 9E10, Santa Cruz), β-tubulin (mAb T4026, Sigma), NACC1 (rAb ab29047, Abcam), ZBTB10 (rAb A303-257A, Bethyl), ZBTB38 (affinity purified rAb prepared by PRIMM), ZBTB4 (rAb #120/4, a gift from Pierre-Antoine Defossez), MORC3 (rAb NBPI-83036, Novus Biologicals rAb), CITED2 (rAb EPR3416(2) Abcam), ZBTB7A (rAb ab123075, Abcam). The sources of antibodies to HSV-1 proteins ICP0 (mAb 11060), ICP4 (mAb 58S) and UL42 (mAb Z1F11) have been described previously [42]. Monoclonal antibody 175 to detect UL6 and rabbit antibody BWp12 for UL12 were kindly provided by Frazer Rixon and Nigel Stow, respectively. Secondary antibodies include horse radish peroxidase conjugated goat anti-rabbit IgG (whole molecule) (Sigma A0545) and goat anti-mouse IgG (whole molecule) (Sigma A4416).

## Supporting Information

S1 Fig Further analysis of cells expressing His-tagged SUMO. A. Infection of HA-HisSUMO2 cells with wt HSV-1 (moi 10) for extended periods, detecting overall reductions in HisSUMO2 conjugates by blotting either for endogenous SUMO2/3 (left) or the His tag (centre). Expression of viral proteins in these samples is shown on the right. B. Over-expression of His-tagged SUMO2 in HA-HisSUMO2 cells. Samples form parental HepaRG or control HA-His Only cells were compared with HA-HisSUMO2 cells for the relative expression of endogenous and ectopic SUMO2 by blotting for the His tag (left) and endogenous SUMO2/3 (right). C. Overexpression of HisSUMO does not cuase substantial changes in the abundance of sumoylated PML. Samples from parental HepaRG and control HA-His Only cells were compared with HA-HisSUMO2 and HA-HisSUMO3 cells by blotting for endogenous PML. The HepaRG lanes were taken from the same exposure of the same blot, eliminating an intervening irrelevant lane. The HA-HisSUMO3 cells have not been used elsewhere in this study. D. Plaque forming efficiency of ICP0 null mutant HSV-1 in parental HepaRG, control HA-His Only and HA-HisSUMO2 cells, determined as described in Materials and Methods.
(EPS)

S2 Fig Summary of an independent quantitative proteomic analysis of total and potential sumoylated protein abundance and SUMO-2 conjugation during HSV-1 infection of HepaRG cells. (A) Schematic overview of the experimental setup, sample preparation and data processing, in this case using only infected (Light) and uninfected (Heavy) samples of HA-HisSUMO2 cells. (B) Overlap of identifications made between the unfractionated cell extract (Crude) and the nickel-purified sample (Pure). (C) Overlap between the proteins IDs in the purified fraction of this double labeled experiment, and those in the triple labeled experiment of Fig 2. (D) Correlation between H/L ratios of the protein IDs in the overlapping fraction of C in the two experiments, divided into likely sumoylated and non-sumoylated substrates on the basis of the H/M ratios in the experiment of Fig 2.
(EPS)

S3 Fig Comparison between ‘Crude’ and ‘Pure’ uninfected:infected protein abundance ratios. (A) The chart shows both total protein abundance and sumoylation changes by comparing log_2_ H/L ratios for proteins from cell lysates (‘Crude’) and from the nickel affinity purified sample (‘Pure’) during HSV-1 infection. The positions of PML, Sp100 and RanGAP are indicated. The chart shows 2222 proteins that had H/L ratios reported in both Crude and Pure fractions in the triple labeled experiment of Fig 2. (B) The table summarizes the data for the three proteins highlighted in A. Protein ID numbers refer to those used in S1 Table.
(EPS)

S4 Fig Expression of selected myc-tagged proteins using HepaRG cells transduced with inducible lentiviral vectors. HepaRG cells expressing the tetracycline repressor and transduced with inducible lentiviral vectors were treated with doxycycline (100 ng/ml) for the indicated times (hours; O/N indicates overnight induction) or left uninduced (-). Whole cell lysates were anlayzed by western blotting using an anti-myc tag antibody. Tubulin serves as the loading control.
(EPS)

S1 Table A complete listing of proteins identified in the triple labeled SILAC experiment. Sheet 1 gives a complete listing of the 6128 protein IDs identified in crude and purified fractions with their basic database entry details. Also included are columns for putative SUMO2 substrate (based on H/M ratios) (column F), if the abundance of sumoylated or total protein forms change significantly during infection, based on H/L and SigB values in the purified and crude fractions respectively (columns G and H), and their predicted molecular weights (column I). Columns M to O present M/L, H/L and H/M ratios for the IDs in crude and purified fractions (NaN implies not detected). Column P notes whether the protein ID was identified previously as sumoylated in [23–26,28,29]. Columns Q and R give SigB values for H/L ratios in crude and pure fractions, and columns S–X give approximate molecular weights of L, M and H versions of the proteins determined by slice-by-slice analysis, while columns Y to AD present the differences of these experimentally estimated molecular weights from the predicted values for that protein ID. Sheet 2 presents a subset of the proteins of sheet 1, including only putative sumoylated substrates. Sheet 3 presents a subset of the protein IDs of sheet 2, sorted for those whose putative sumoylated forms in the purified fraction change significantly during infection, based on SigB <0.1. Sheet 4 presents a subset of the proteins IDs of sheet 1, sorted for those whose total amounts in the crude fraction change significantly during infection, based on SigB values of <0.1. The lists include multiple entries for proteins such as PML for which several different isoforms were detected but they have not been manually edited to remove any duplicate entries.
(XLSX)

S2 Table Identification of viral proteins in crude and purified fractions. Columns A to E give database proteins IDs, gene names, proteins names, predicted molecular weights and sequence lengths of all viral proteins identified in crude or purified fractions, or both. PEP is MaxQuant protein posterior error probability. Columns G–I gives total peptide intensity values for each protein based on extraction ion chromatograms. Columns J and K give the apparent molecular weights in the crude and purified fractions, based on peak gel slice analysis, columns L and M present the differences in these values from the predicted values, and columns N and O note whether the protein is heavier the predicted in the crude fraction. Columns P to X and Y to AG present the intensity values in the slice-by-slice analyses in the crude and purified fractions respectively, while columns AH to AP and AQ to AY present the proportions of the total amount of protein found in each gel slice for the crude and purified fractions.
(XLSX)

S3 Table A comparison of all protein IDs detected in both the triple and double labeled experiments. The triple and double labeled experiments were as summarized in Figs 2 and 3 respectively, from which there were 6280 proteins in common. Columns A to I are as for columns A to I in S1 Table. Columns J to M note whether the proteins were detected in both H and L fractions of the purified and crude fractions of the double and triple labeled experiments (and hence give an H/L ratio), columns N and O present the normalized values of these ratios in the double labeled experiment, while columns P to U give the normalized M/L, H/L and H/M ratios for the crude and purified fractions of the triple labeled experiment. Column V notes whether the protein ID was identified previously as sumoylated in [23–26,28,29]. Columns W to AP give apparent molecular weights derived from slice-by-slice analysis of the crude and purified fractions of both experiments, and the differences for these values from the predicted in the H, L and M (triple labeled experiment only) samples. Columns AQ to AT present SigB values of H/L ratios of proteins in the crude and purified fractions of the double and triple labeled experiments. A graphical representation of the correlation of these data from the double and triple labeled experiments is presented in S2D Fig.
(XLSX)

S4 Table Proteins with H/L ratios of 2 or greater in the purified fraction of the double labeled experiment which were not recorded as significantly regulated in the triple labeled experiment. Columns A to I are as for S1 Table columns A to I. Columns J to Q give the normalized M/L, H/L and H/M ratios for the crude and purified fractions of the double and triple labeled experiments, as noted. Column R notes whether the protein ID was identified previously as sumoylated in [23–26,28,29]. Columns S to V give the SigB values of the H/L ratios in the crude and purified fractions of the double and triple labeled experiments. Columns W to AF give the apparent molecular weights of the protein IDs in H and L samples of the crude and purified fractions of both experiments (based on slice-by-slice analysis), while columns AG to AP give the differences between these and the predicted values.
(XLSX)

S5 Table Proteins not defined as potentially sumoylated which have H/L ratios of greater than 2 in the purified fraction. Column headings are as for S1 Table.
(XLSX)

S6 Table Putative SUMO2 substrates whose H/L ratios are reduced in both purified and crude fractions. Column headings are as for S1 Table.
(XLSX)

S7 Table Proteins with significantly changed H/L ratios in the crude sample. Excluding regulated putative SUMO2 substrates. Column headings are as for S1 Table.
(XLSX)

S8 Table Cellular proteins whose degree of sumoylation appears to increase during infection. Column headings are as for S1 Table.
(XLSX)

S9 Table Cellular proteins that increased in overall abundance during HSV-1 infection. Column headings are as for S1 Table.
(XLSX)
